# Calculating a Phase Diagram of a Simple Water Model Using Unsupervised Machine Learning on Simulation Data

**DOI:** 10.1021/acs.jctc.4c01456

**Published:** 2025-04-14

**Authors:** Peter Ogrin, Tomaz Urbic

**Affiliations:** Faculty of Chemistry and Chemical Technology, University of Ljubljana, Vecna Pot 113, SI-1000 ljubljana, Slovenia

## Abstract

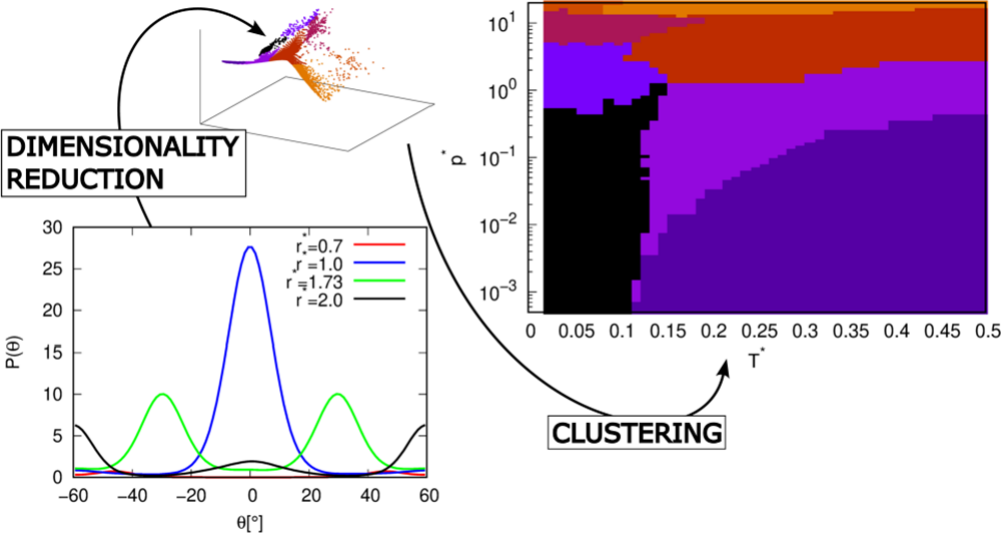

We use unsupervised machine learning to construct a phase diagram of a simple 2D rose water model. The machine learning method that we use is a combination of dimensionality reduction methods and clustering algorithms. Two different data sets from the same simulations are used as input data for machine learning. These are angular distribution functions and a set of different thermodynamic, dynamic, and structural properties. To evaluate the efficiency of the method, the machine learning results are compared to manually determined phase diagrams. We show that the methods successfully predict the phase diagram of the rose water model. Furthermore, the phase diagrams obtained from the two data sets are in semiquantitative agreement with each other. Four different solid phases, one liquid phase, and one gaseous phase were determined. The method we have presented is straightforward and easy to implement. It requires almost no prior knowledge of the system to obtain a phase diagram. The method can also be used to distinguish between the different parts of the same phase that have different properties or a sufficiently different structure, and in this way find local differences and anomalies.

## 1 Introduction

Water is a very abundant substance on Earth, and its presence in systems is often crucial to the properties of the system. It plays an important role in many living and nonliving systems, where it acts as a solvent or even as an active part of chemical reactions. For all known organisms, water is the only substance that makes life possible, as it is a solvent in which biochemical reactions take place. In addition, water is also crucial for the structure and thus function of proteins. Many of the properties that make water unique and indispensable are anomalous properties. Such anomalous properties are, for example, the higher density of the liquid phase compared to the solid phase, the high heat capacity, the negative thermal expansion coefficient, etc. One of the interesting anomalous properties of water is also the rich and complex phase diagram,^1,2^ which shows 20 different solid ice phases.^3^ The experimental studies of the phase behavior of water are important because it is the easiest way to obtain information about real systems. However, nowadays, when computer technology is improving incredibly quickly, computational methods have become indispensable in virtually all branches of science. With the help of computational methods, we can obtain information much faster than with experimental methods and at the same time obtain more in-depth information and relationships between different pieces of information.

When the computational methods are used to investigate water phases, the phase diagram naturally depends on the water model used. The phase diagrams of popular water models from the SPC and TIP groups have already been determined.^4−9^ For the TIP3P model, only two different solid phases were found in the phase diagram,^10^ which is quite poor compared to the experimental 20 solid phases. A slightly better phase diagram is the phase diagram of the SPC/E model, as it shows five different solid phases.^5^ The model also predicts a reentrant behavior of water (i.e., a change of sign in the slope of the melting curve in the p-T diagram).^5^ Another model that also exhibits reentrant behavior is TIP4P, which also shows a more accurate phase behavior, as its phase diagram qualitatively matches the experimental phase diagram. In addition, the model can form 7 stable solid phases.^9^ The main difference between the TIP4P phase diagram and the experimental phase diagram lies in the position of the phase transition from a liquid to high-density ice. To further improve the prediction of the phase behavior of water, the TIP4P model was reparameterized in the TIP4*P*/2005 model. The reparametrization is based on the stability of the different ice phases, and so it is not surprising that the model is in semiquantitative agreement with the experimental water in terms of the phase transitions between the different ice phases.^11^ However, there is a problem with TIP4*P*/2005, namely, the obviously wrong position of the solid–liquid phase transition, as it occurs at too low temperatures and too high pressure. On the other hand, TIP4*P*/2005 is very successful in predicting the liquid–vapor coexistence line and also the critical point, as both are in very good agreement with the experiments.^10^ A later recalculation of the phase diagram of TIP4*P*/2005 was performed by using direct coexistence simulations. The results showed that the stability of ice III is different from what was thought before.^6^ While the reparametrization of TIP4P in TIP4*P*/2005 improved the accuracy of the behavior of the solid phases, a similar improvement was not observed when an additional interaction point was added to the model. This is because TIP5P does not describe the phase behavior of ice phases as successfully as it predicts only three solid phases. All in all, classical realistic models can reproduce the phase behavior of water very well if the parametrization of the model is based on data that contain sufficient information about the desired phase behavior. On the other hand, with advances in computer technology, increasingly complex models and methods can be used, leading to more detailed and accurate phase diagrams. With the computing speed achievable today, quantum mechanical methods can also be used to calculate the phase diagrams of water.^12,13^

While technology is improving and allowing us to perform more complex and time-consuming calculations, simple water models are still used. The advantage of simple models is that they allow a more in-depth investigation of the physical causes and mechanisms behind different phenomena (e.g., the hydrophobic effect), as the number of models that affect the phenomena under investigation is reduced. Moreover, simple models can serve as testing grounds for different methods ranging from statistical mechanical theories to machine learning methods. In this way, we also used the model used in this work. Here we used the Rose water model, a simple water model with explicit hydrogen bonding potential.^14^ The simplicity of the model allowed us to develop a machine learning approach for phase diagram determination, which was developed in this work. For example, due to the two-dimensionality of the model, we could more easily recognize the differences between different phases based on the snapshots of the system. The Rose model is a model similar to the Mercedes-Benz water model,^15,16^ the model replicates many of the anomalous properties of water and has already been studied with different statistical mechanics theories and simulations.^17−23^

There are many methods used to determine phase transitions and phase diagrams, including methods such as Maxwell construction, thermodynamic integration,^24,25^ different modifications of molecular dynamics,^26^ Monte Carlo simulations in Gibbs ensemble,^27^ Gibbs–Duhem integration^28^ and nested sampling algorithm.^29−32^ Nested sampling is a good alternative to MD and MC simulations, but it is not such a powerful technique to determine all phase transitions without much effort. In our previous work, we used the nested sampling algorithm to determine the phase diagram of the Mercedes Benz water model.^33^ The algorithm allowed us to calculate the properties of the model over a wide range of conditions, and from the extrema of the thermodynamic fluctuation functions, we were able to determine the positions of some phase transitions. However, we were not able to determine all phase transitions in this way. Therefore, a more detailed investigation of the properties of the system under different conditions was required to further differentiate the phases.

To automate the process of discovering phase transitions, we turned to machine learning. In recent years, machine learning has become increasingly popular in different areas of industry and science. Roughly speaking, machine learning methods can be divided into supervised and unsupervised machine learning. When it comes to determining phase transitions and phase diagrams, both types of machine learning methods can be used. To start with supervised machine learning: Convolutional neural networks have been successfully used to study phase transitions of different lattice systems.^34−38^ In addition, neural networks have also been used to study the phase behavior of off-lattice systems.^39−43^ Neural networks were used to study water–alcohol mixtures modeled with a continuous shoulder well model.^44^ The neural networks were able to predict the fluid phases and solid phases of pure CSW and CSW-alcohol mixtures. Although supervised machine learning methods, such as neural networks, are very powerful and can be applied to various problems, the problem with supervised machine learning is that it requires training sets on which the model is trained. In other words, we must already have an outcome on which to train the model. The bridge between supervised and unsupervised machine learning is semisupervised machine learning,^45^ which can be used when you have a lot of unlabeled data and at the same time have some labeled data. On the other hand, there is unsupervised machine learning, which ideally requires no prior knowledge of the system. Unsupervised machine learning can be further divided into shallow and deep methods. An example of a shallow method is the widely used principal component analysis,^46^ which was successfully used in the investigation of the phase behavior of the Ising model.^47,48^ An example of a deep method is autoencoders, which have also been used to study the phase behavior of different lattice and off-lattice systems.^48−53^ Focusing on off-lattice systems, an unsupervised ML algorithm was developed to differentiate the local environment of a colloidal system using a neural network-based autoencoder in combination with Gaussian mixture models.^52^ Vectors of bond orientation order parameters were used as input data to the ML algorithm. The algorithm successfully identified relevant local environments. Later, the same unsupervised ML algorithm was used to identify structural heterogeneities in three archetypal glass formers.^53^ Neural network-based autoencoders combined with Gaussian mixture models were also used to study the crystal growth of different ice phases,^54^ with data coming from MD simulations. The authors discovered that the growth rate of ice VII is faster due to the presence of a plastic ice layer on the surface of ice VII, indicating that translational and rotational orders are decoupled. Principal component analysis (PCA) has been successfully used to detect the freezing transition of two-dimensional hard disks, three-dimensional hard spheres, and the liquid–gas phase transition of a patchy colloid model.^55^ It was also shown that PCA is able to detect order-parameter-like quantities that correlate with phase transitions. The method was further developed and used to explore phase transitions of hard ellipses and demixing transition in binary Widom-Rowlinson mixtures.^56^ In combination with the diffusion map, PCA was also applied to distinguish between different states of polymer configurations.^57^ Unsupervised (and supervised) ML was also used to investigate structural transitions of polymers into polymer–nanotube composites.^58^ The authors concluded that the use of ML also reduces the likelihood of human error and the need for human intuition in recognizing boundaries between configurational phases.

In this work, we used unsupervised machine learning methods to determine the phase diagram of a simple water model. The approach used is a combination of dimensionality reduction methods and clustering algorithms. First, the approximate phase diagram of the rose water model was manually constructed from the simulation data, as we did for the MB water model in our previous study.^33^ The manually constructed phase diagram served as a reference against which we compared the ML results. Then the unsupervised ML was applied to the simulation data to obtain the phase diagram of the water model. Two sets of simulation data were used. First, the phase diagram was determined based on angular distribution functions at different radial distances. In the second part, various (one-dimensional) thermodynamic, static, and dynamic quantities from MD simulations were used to determine the phase diagram.

## 2 Model and Method

### 2.1 Model

In this work, we have examined the phase diagram of the Rose water model.^14^ This is a simple two-dimensional water model in which the molecules are represented by Lennard-Jones discs, with an additional hydrogen bonding potential for the hydrogen bonds between the molecules:
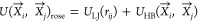
1where *r*_*ij*_ represents the distance between the centers of the molecules *i* and *j*,  and  are vectors of the positions and orientations of the molecules *i* and *j*. The LJ potential has a common form:
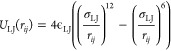
2The two molecules that are in pair interactions both contribute to the total energy of the interaction, while the contribution of one molecule is independent of the contribution of the other molecule:
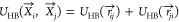
3Due to this independence, a molecule can form “half” hydrogen bonds, i.e., a situation in which one molecule is favorably oriented to form a hydrogen bond while the other molecule is unfavorably oriented to form a hydrogen bond. Although the formation of “half” bonds is possible, it is energetically less favorable than a normal hydrogen bond, so the full hydrogen bond is usually formed when this is possible. Another situation occurs when the other molecule in the pair cannot form a hydrogen bond. In such a situation, a half bond will always form, as it is more favorable than an LJ contact.

The energy contribution of one molecule to the hydrogen bonding potential is a product of two terms, the first of which depends on the orientation (*U*(θ_*ij*_)) and the second on the distance (*s*(*r*_*ij*_)).
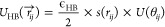
4where  is a vector between the molecules *i* and *j*, which is oriented according to the body frame of the molecule *i*, *r*_*ij*_ is the length of the vector, and θ_*ij*_ is the orientation angle of the vector in the body frame of the molecule *i*. The HB energy parameter, which represents the maximum energy of the hydrogen bond, is ϵ_HB_.

The energy contribution of the orientation of molecule *i* described by the orientational term of the HB potential depends on the orientation of molecule *i* with respect to the position of molecule *j*, or from the perspective of molecule *i*, it depends on the position of molecule *j* with respect to the body frame of molecule *i*. The orientational term consists of a combination of sine functions known as the rose function. In the case of our model, we use the 3-petal rose function:
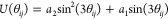
5where *a*_1_ and *a*_2_ are coefficients that determine the shape of the potential with respect to the angle. The use of the rose function also gave the model its name. For easier use in the simulations. The same function can be rewritten in the Cartesian coordinate system:
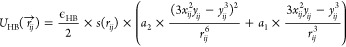
6where *x*_*ij*_ and *y*_*ij*_ are Cartesian coordinates of the molecule *j* in the body frame of the molecule *i* and .

In the ideal case, when the interacting molecules are in the position where the interaction between them is the strongest (this is the case when the arms of the potential are aligned with the centers of the molecules and are also parallel), the value of the orientational term is −1. To normalize the interaction energy of the entire interaction,  is therefore substituted intoeq 4.

To model the distance dependence of the interaction, the double-sided cubic switching function is used as the distance-dependent term:
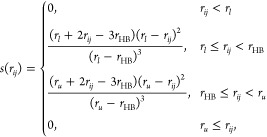
where *r*_HB_ represents the HB distance, *r*_*l*_ and *r*_*u*_ correspond to the lower and upper limits of HB. The switching function is symmetrical because |*r*_HB_ – *r*_*l*_| = |*r*_HB_ – *r*_*u*_| = *r*_fwhm_, where *r*_fwhm_ is the “full width at half-maximum” of the peak formed by the function.

**Figure 1:**
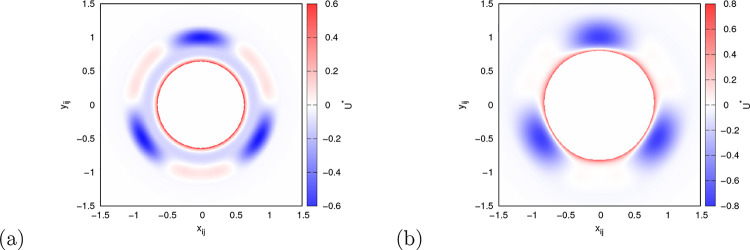
Potential energy surfaces of the rose water model with (a) MB parametrization and (b) real parametrization.

In this work, we utilize two different parametrizations of the rose model. To illustrate what the model looks like and what differences there are between the two parametrizations, the potential energy surface of both parametrizations of the model is shown in Figure 1. The rose model was originally developed as a representation of the Mercedes-Benz (MB) water model, and the first parametrization is chosen to closely match the properties of the MB model, as outlined in previous studies.^15,16^ This first parametrization is referred to as the MB parametrization throughout the text. The parameters for this model are as follows: ϵ_LJ_ = 0.1, σ_LJ_ = 0.7, ϵ_HB_ = 1, *r*_HB_ = 1, *r*_fwhm_ = 0.2, *a*_1_ = 0.6, and *a*_2_ = – 0.4. The Lennard-Jones potential in this parametrization is identical to that in the MB model. The second parametrization, hereafter referred to as the real parametrization, was developed to address certain exaggerations present in the MB parametrization and to make the model’s properties more realistic. Specifically, the distances between interacting particles are adjusted so that the minimum of the Lennard-Jones potential coincides with the length of the hydrogen bond. Additionally, the Lennard-Jones interaction is strengthened compared to the MB parametrization. The key differences in the real parametrization are ϵ_LJ_ = 0.2 and σ_LJ_ = 0.890899 and a larger hydrogen bond width, with *r*_fwhm_ = 0.41666. All other parameters remain unchanged from the MB parametrization. The details of how the parameters of these two parametrizations were determined can be found in the following articles.^14,17^

### 2.2 Nested Sampling Method

Using the nested sampling algorithm at constant pressure and flexible boundary conditions, a sequence of decreasing configurational enthalpy levels, *H*_*i*_, (*H* = *U* + *pV*), is constructed. Each volume of the configuration space χ_*i*_ is bounded from above by each enthalpy level such that χ_*i*_ is approximately a constant factor smaller than χ_*i*–1_ of the level above. If we combine the sequence of enthalpy levels, we obtain the cumulative density of states χ(*H*).^29,30^ Once the cumulative density of states has been obtained, the constant pressure partition function can be calculated as follows
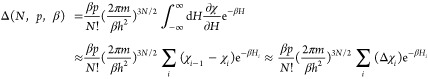
7where *N* represents the number of particles of mass *m*, *h* is Planck’s constant. The sequence of enthalpies and configuration space volumes is independent of the temperature; therefore, the partition function can be calculated for any temperature after the configuration space has been sampled, since the sequence of enthalpies and the corresponding configurational space volumes are independent of the temperature.

The following is a brief description of the nested sampling algorithm. Initially, a set of *K* random configurations is generated, and then, the following loop is run, starting with *i* = 1. (I.) The enthalpy of all samples is calculated, and then the new enthalpy limit, *H*_lim_ ← *H*_*i*_, is set to the enthalpy of the sample with the highest enthalpy. While χ_*i*_ = χ_0_(*K*/(*K* + 1))^*i*^, where , is the corresponding phase space volume with an enthalpy equal to or less than *H*_lim_. The simulation cell is limited to a volume smaller than *V*_0_, as the sampling must take place from a compact configuration space. However, the volume is large enough to almost correspond to an ideal gas, i.e., *k*_B_*T* ≪ *pV*_0_. (II.) In the next step, we remove the sample with enthalpy *H*_*i*_ from the set and generate a new random configuration within the configuration space, also taking into account that the enthalpy of the new configuration must be smaller than *H*_lim_. The new configuration can be generated by performing a random walk with a length of *L* steps on a clone of a randomly selected existing configuration, which is in the current enthalpy limit. In other words, an existing configuration is randomly selected and duplicated. Within the duplicated configuration, a particle is then randomly selected and randomly moved so that the enthalpy of the configuration is lower than the enthalpy limit. The random selection of the particle and the movement represent 1/*N* of a step, which means that this is repeated *L* × *N* times, meaning that each particle is moved *L* times on average. The change in volume is also attempted once per step. The algorithm has some similarities with Monte Carlo simulation, except that the enthalpy of the newly generated configuration must always be lower than the enthalpy limit. (III.) Finally, we leave *i* ← *i* + 1 and then return to step (I.).^29,30^

Once we have obtained the partition function, we can use it to calculate various thermodynamic quantities.

NS simulations were performed at different pressures to capture the conditions of the entire phase diagram. To locate the possible phase transitions, the temperatures of the extrema of the thermodynamic functions, such as the heat capacity, were then determined.

The details of the NS simulations are as follows: The number of particles used was 32, the number of configurations was set to *K* = 500, the number of random walk steps was *L* = 5000, and the number of iterations was 500,000.

### 2.3 Molecular Dynamics Simulations

We also used MD simulations to calculate the properties of the rose water model, which were then used in further calculations. The simulations were performed with the in-house MD code,^59^ the code was originally developed for the MB water model, while for the purposes of this work, we have changed the interaction potential to the rose water model. The simulations were performed in the NPT ensemble. To mimic the macroscopic system, periodic boundary conditions and a minimal image convention were used. For the trajectory integration, the Velocity Verlet integrator^60^ was used, with a time step length of 0.001 . The system has been equilibrating for 100,000 steps. This was followed by the sampling phase, in which various structural, thermodynamic, and dynamic variables were sampled. The sampling phase was carried out in 20 series, each series being 100,000 steps long. There were 200 water molecules in the system throughout the simulation. The initial configuration of the molecules was randomly generated, ensuring that the molecules did not overlap. A simple velocity rescale was used as the thermostat in the equilibration phase of the simulation, and stochastic velocity rescaling^61^ was used later in the sampling phase. For the barostat, we used the Berendsen barostat^62^ in the equilibration phase, while the stochastic cell rescale^63^ was used in the sampling phase. The coupling constant for thermostats was 0.01 and for barostats, it was 0.1.

Thermodynamic, structural, and dynamic properties were calculated using standard relations. The angular distributions were calculated by counting the molecules at different positions and plotting them in the histogram.

### 2.4 Dimensionality Reduction and Manifold Learning

Manifold learning, also known as nonlinear dimensionality reduction, is an umbrella term for techniques that seek projections of high-dimensional data onto low-dimensional latent manifolds. One technique that looks for low-dimensional projections of high-dimensional data and is probably the most widely used is principal component analysis (PCA).^46^ PCA is a linear dimensionality reduction technique that reduces the dimensionality of the data by transforming the data into lower dimensions represented by principal components that are orthogonal to each other and describe as much variance as possible. However, PCA also has its limitations. The most obvious one is that the underlying structure of the data should be linear.^64^ Manifold learning techniques can be seen as generalizations of linear dimensionality reduction techniques such as PCA. Below is a brief description of the manifold learning techniques we use. The techniques are well-known and widely used, so you can find more detailed descriptions in the literature.

#### 2.4.1 MDS

Multidimensional scaling (MDS)^65,66^ is a technique that creates a representation of data in low-dimensional space while attempting to maintain the pairwise distance between data points in the original high-dimensional data as closely as possible. There are two types of MDS, namely metric and nonmetric MDS. In this work, the metric MDS was used.

#### 2.4.2 Isomap

Isomap or isometric mapping^67^ is a technique that calculates a low-dimensional embedding of high-dimensional data in such a way that the geodesic distances between all points are preserved. Isomap is a kind of extension of MDS, but it offers more flexibility in learning various nonlinear manifolds. While MDS searches for the shortest Euclidean distance between two data points in high-dimensional space, the isomap searches for geodesic distances and can therefore be more successful in finding intrinsic low-dimensional geometry in the original data.

#### 2.4.3 Spectral Embedding

Spectral embedding^68^ is a technique that uses Laplacian eigenmaps to find a representation for data lying on a low-dimensional manifold embedded in a high-dimensional space.^68^ The low-dimensional representation of the data is computed using a spectral decomposition of a graph Laplacian, which preserves the local neighborhood distances between the data points.

#### 2.4.4 t-SNE

T-SNE or t-distributed stochastic neighbor embedding was developed to visualize high-dimensional data in a two- or three-dimensional map. The technique is based on stochastic neighbor embedding, using the Student’s t-distribution for embedding.^69^ For each pair of data points, the algorithm constructs a Gaussian probability distribution in the original high-dimensional space. The probabilities are then transformed into the low-dimensional space, where they are represented by Student’s t-distributions. In contrast to the Isomap, t-SNE focuses more on the local structure of the data. The most appropriate distributions are found by minimizing the Kullback–Leibler divergence of the joint probabilities in the original and embedded space.

### 2.5 Clustering Algorithms

After dimensionality reduction of the analyzed data, we used clustering algorithms to group data points that are similar into clusters with similar properties. In our case, these clusters were intended to represent different phases of the water. However, in some cases, some phases were separated, and others were not. In addition, there are also examples where the algorithms identified multiple phases where we expected only one. Below is a brief description of the clustering algorithms we used in this work. The algorithms are widely used and well-known, so interested readers can refer to the literature for a more detailed description.

#### 2.5.1 K-Means

K-means clustering is a technique that clusters data points into K clusters of equal variance such that each point belongs to the cluster with the nearest centroid (cluster center). When clustering, the algorithm minimizes the variance within the clusters, which is called inertia. The number of clusters K is usually determined in advance. The k-menas was first mentioned in 1967.^70^ The original method has several disadvantages; for example, due to inertia, the clusters are assumed to be convex and isotropic. Another disadvantage is that the result strongly depends on the initialization of the centroids. To overcome this problem, k-means++ was introduced, which uses a randomized seeding procedure and therefore significantly improves both the speed and accuracy of k-means.^71^

#### 2.5.2 Ward Hierarchical Clustering

Hierarchical clustering is probably the most intuitive type of clustering. In hierarchical clustering, the algorithm groups the samples hierarchically into clusters. In agglomerative clustering, each data point starts as a separate cluster, and then the algorithm combines pairs of clusters according to the hierarchy of distances between the pairs. The results of this clustering can be represented as a dendrogram.^72^ Several linkage criteria can be used to decide which clusters should be merged. In this work, we have used the Ward linkage.^73^ The Ward linkage looks for a minimum variance of points within a cluster to join the clusters. The distance between data points can be measured with different metrics; we used normalized Euclidean distances.

#### 2.5.3 DBSCAN

DBSCAN or density-based spatial clustering of applications with noise is a technique that clusters data points based on the density of points in space such that clusters are assigned to areas of high point density separated by areas of low density.^74^ The algorithm requires two main parameters, namely, the minimum number of neighbors of the point to be considered as the core point and the distance to the core point that must lie within these neighbors. When applying this clustering method in this work, we tried many combinations of the two parameters and chose the values of the parameters so that the number of points that did not belong to any cluster was as small as possible, and the clustering result appeared meaningful.

## 3 Results and Discussion

All results are shown as reduced units. HB energy parameter ϵ_HB_ is used to normalize temperature and excess internal enthalpy (A* = *A* |ε_HB_|, T* = *k*_B_∗*T* |ε_HB_|), distances are normalized with the characteristic length of the hydrogen bond *r*_HB_ (r* = *rr*_HB_). Pressure in reduced units is defined as .

**Figure 2:**
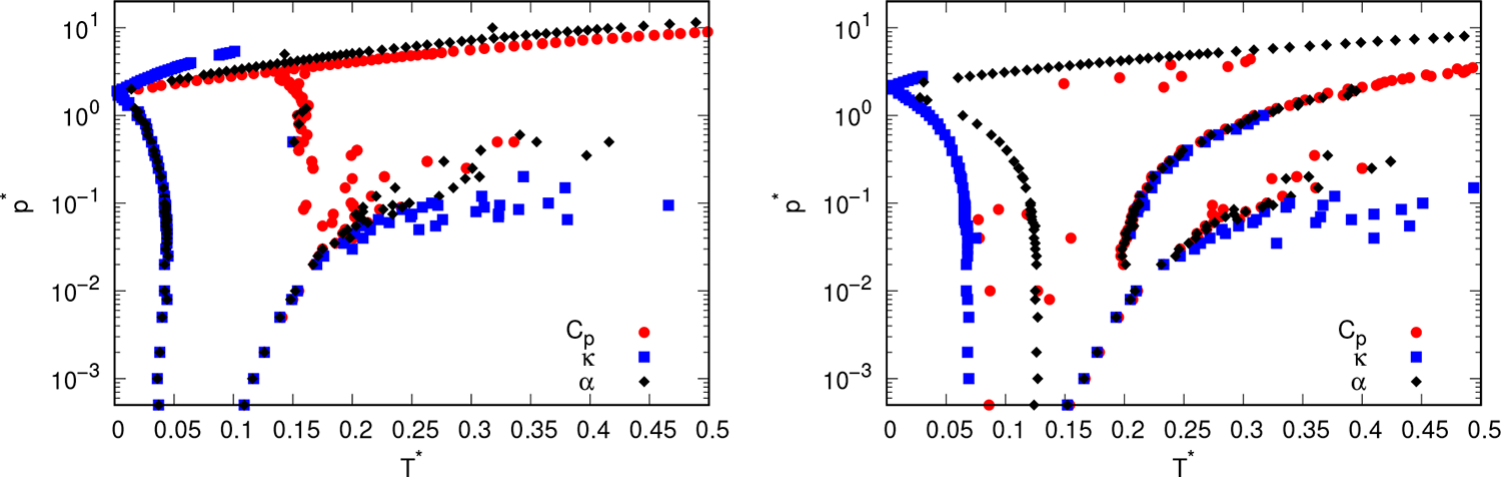
Pressure–temperature phase diagram of rose model with MB parametrization (left) and real parametrization (right) as obtained from different thermodynamic properties calculated using nested sampling. The points represent extrema in different quantities: red circles represent phase transitions obtained from heat capacity, blue squares are phase transitions from isothermal compressibility, black diamonds are phase transitions obtained from thermal expansion coefficient. 32 particles were used in nested sampling.

In our previous article,^33^ we used thermodynamic data from nested sampling and structural data from MD simulations to determine a phase diagram of the Mercedes-Benz water model. In this work, we used unsupervised machine learning methods to extract information about the phase diagram of the rose water model. To start and to have some kind of reference with which to compare the machine learning results, we first used the same methods to obtain the phase diagram of the rose model as we did previously for the MB model. First, the thermodynamic properties of the rose model were calculated with MB and real parametrization for different temperatures and pressures. Then the extrema of the heat capacity, isothermal compressibility, and thermal expansion coefficient were determined, as they usually indicate the presence of a phase transition. Figure 2 shows the extrema of the aforementioned thermodynamic quantities for both parametrizations of the model. Note that not all phase transitions could be found with this method and that, due to the numerics of the algorithm, the vertical lines at low temperatures could be an artifact of the numerics. Nonetheless, the results of the nested sampling give us a solid estimate of where some phase transitions are located.

### 3.1 Determining Phases from Angular Distribution Functions

#### 3.1.1 MB Parametrization: Manual Determination

Next, we use the same procedure as before for the MB model to obtain a more detailed phase diagram of the rose water model. The angular distribution functions at different radial distances between the water molecules were analyzed. The number of peaks in each angular distribution function was determined for every phase point, and from these numbers, the approximate phase diagram was obtained. Basically, we manually looked at the angular distribution functions (and snapshots) at different conditions and decided under which conditions they change so much that there could be a phase transition. During the procedure, we also took into account the diffusion coefficient, which tells us which phase is solid, liquid, or gas. For a more detailed description of the procedure, the reader should read our previous study.^33^ First, the rose water model with MB parametrization is analyzed as it has more different phases than the real parametrization of the model. The reason is that the MB parametrization has two different radial distances for the LJ contact and the hydrogen bond, while these two distances are the same in real parametrization.

**Figure 3:**
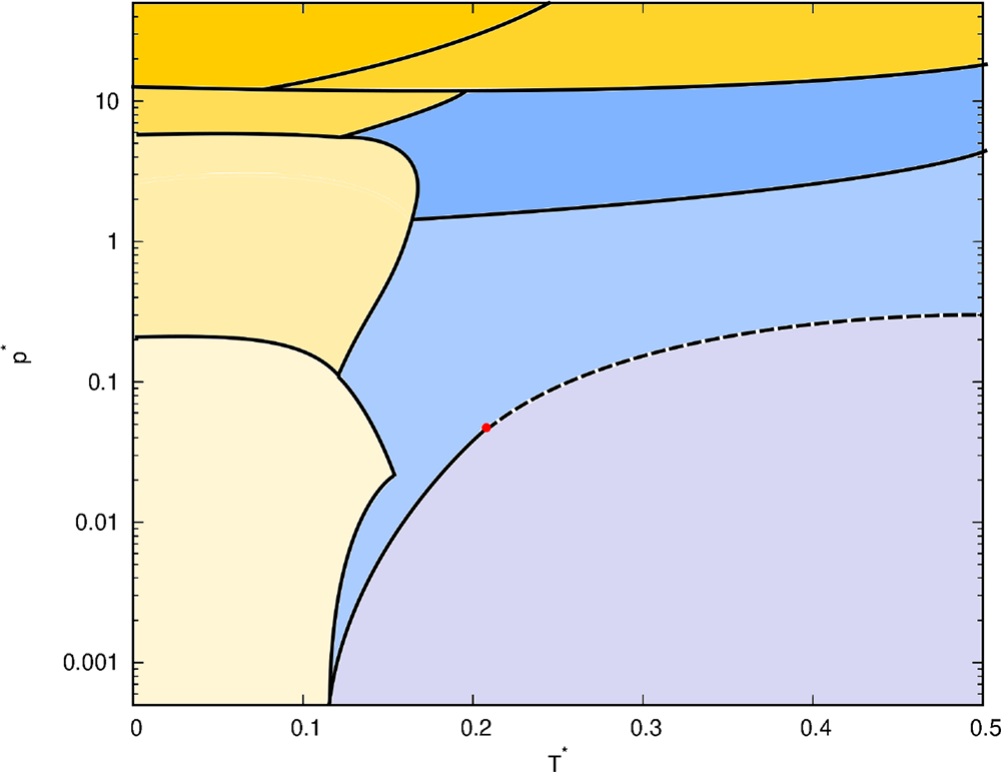
Pressure–temperature phase diagram of rose model with MB parametrization obtained from angular distribution functions by counting the number of the peaks in the functions. The diffusion data from simulation helped us to locate the liquid phases.

In Figure 3, the phase diagram of the rose water model with MB parametrization is shown. The diagram was determined using the method just presented. Note that the positions of some phase transitions are less reliable than others because this phase diagram was determined manually by combining different results. For example, the angular distribution functions change continuously between some phases, and there is no sudden jump-like change in the distribution. Therefore, one has to decide where to draw the exact boundary between two phases. Nevertheless, this phase diagram provides a good estimation of what a phase diagram of the rose water model with MB parametrization looks like. Below is a brief description of the phases, accompanied by figures of the angular distribution functions and snapshots of the system.

**Figure 4:**
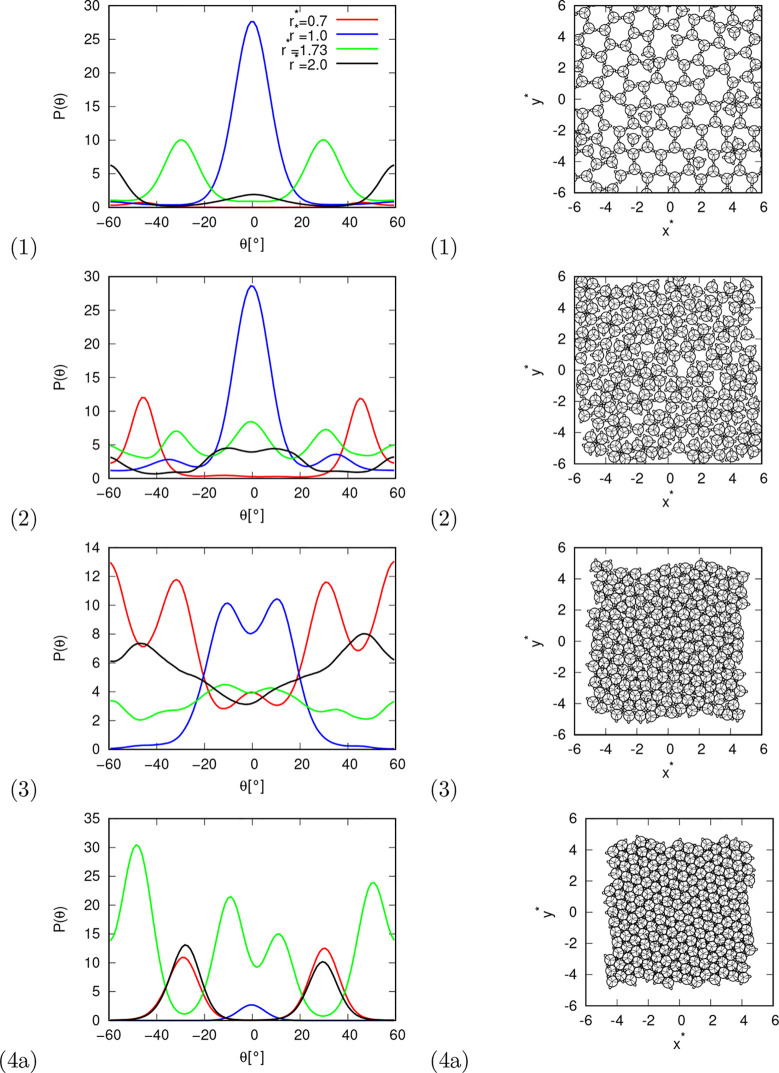
Angular distribution function between HB arm and line connecting centers of molecules at selected distance (left column), and snapshots of the system. Distributions and snapshots are shown for different phases at conditions: (1) p* = 0.01, T* = 0.10, (2) p* = 1.0, T* = 0.06, (3) p* = 8.0, T* = 0.05, and (4a) p* = 20.0, T* = 0.06.

**Figure 5:**
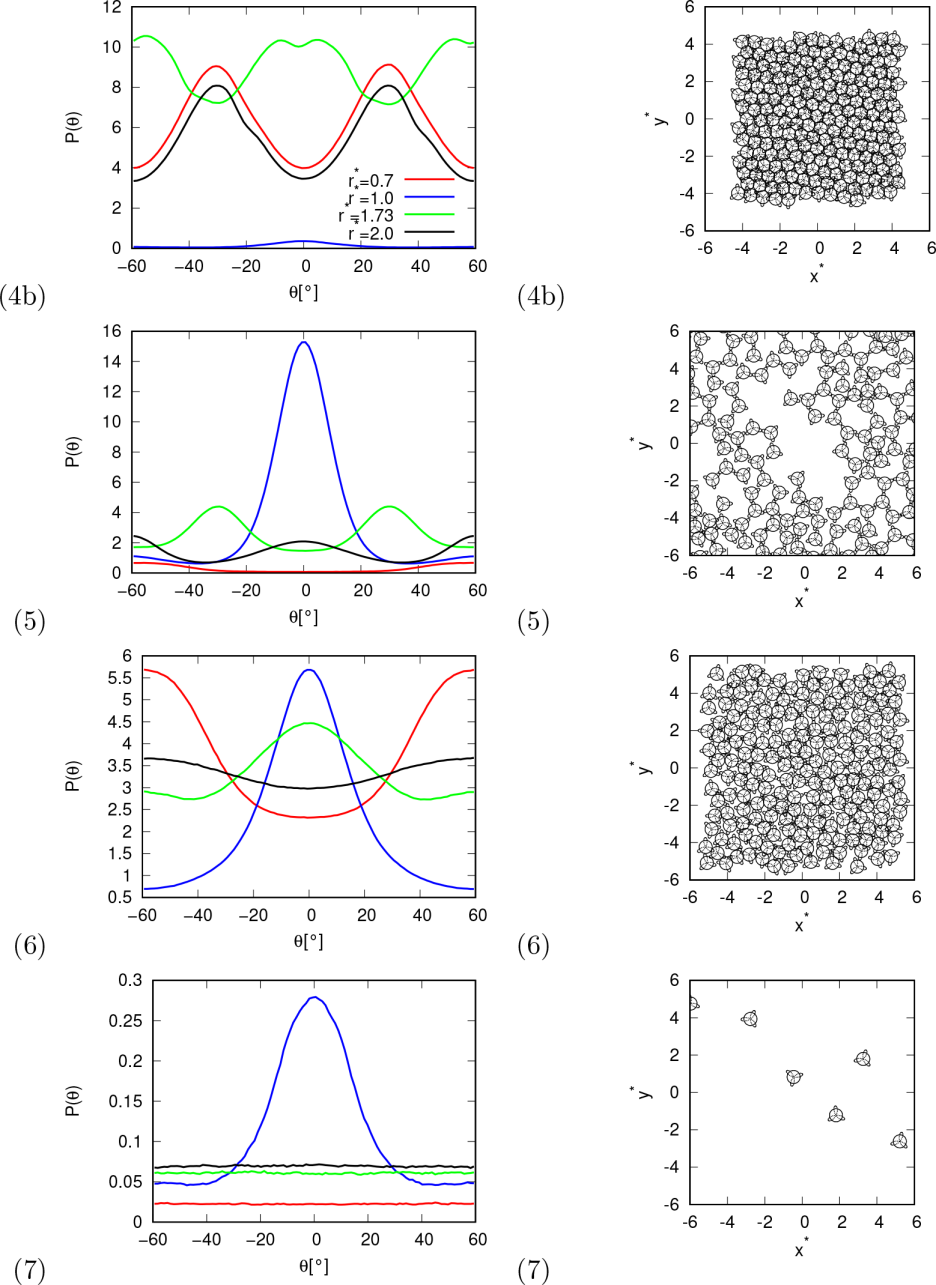
Angular distribution function between HB arm and line connecting centers of molecules at selected distance (left column), and snapshots of the system. Distributions and snapshots are shown for different phases at conditions: (4) p* = 20.0, T* = 0.25, (5) p* = 0.01, T* = 0.15, (6) p* = 4.0, T* = 0.25, and (7) p* = 0.01, T* = 0.30.

In Figures 4 and 5, angular distribution functions and snapshots of the system are shown for different phases of the rose water model with MB parametrization. Starting with phase (1) (Figure 4 (1)), this is a solid phase in which the particles form a hexagonal crystal in which the molecules are connected by hydrogen bonds. This phase occurs at low temperatures and pressures. In the angular distribution functions, this can be seen as a large peak at a radial distance of 1.0° and an angle of 0°, which corresponds to a direct hydrogen bond. The two peaks are at a radial distance of 1.73 and angles of −30° and +30°, which correspond to the two molecules that are connected with HB to the same connecting molecule.

Next is phase (2), which is also a solid phase, but its structure is not as ordered as that of phase (1). This phase occurs at similar temperatures to phase (1), but at a higher pressure. No distinguished structural pattern is visible to the naked eye in the snapshot. From the angular distribution function at a distance of 1.0, we can see that there are still many molecules that are directly connected by hydrogen bonds. The height of the peaks in the angular distribution at a distance of 0.7 (which corresponds to a direct LJ contact) increases significantly. The most striking peaks at a distance of 0.7 are at angles ±45°, which corresponds to a quadruple of particles where two molecules are connected by HB and form a cross.

At similar temperatures and even higher pressures, the next solid phase is present (3). From the snapshot, it can be seen that this phase is relatively dense, as there are almost no vacancies. However, the packing of the phase is not a hexagonal dense packing, as it is rather disordered. The angular distribution function at a distance of 1.0 shows a splitting of the peak at an angle of 0° into two peaks. Angular distribution functions at a greater distance have less pronounced peaks, which indicates a loss of the long-range orientation order.

The last solid phase is phase (4), which occurs at high pressures over the entire temperature range investigated. This phase exhibits hexagonal dense packing and is more ordered than the solid phase at a lower pressure, which is also evident from the angular distribution functions. In Figure 3, this phase is divided into two parts, as the results of the machine learning algorithms (presented later in the article) indicate that the two parts are clearly different from each other. The low-temperature part a) of this phase is much more structured and ordered than the high-temperature part (4b). However, the difference in order is probably due only to the temperature difference and not to the phase transition.

The next phase (5) is a liquid phase. It is similar to solid phase (1) in the sense that part of the hexagonal HB lattice is still preserved. We could say that phase (5) is a liquid version of phase (1). Most of the molecules are still connected to the HB, which is also reflected in the angular distribution functions. In contrast to phase (1), phase (5) is more disordered.

Phase (6) is also a liquid phase. As can be seen from the snapshots, this phase is much denser than phase (5) and much less ordered. By some criteria, there is basically only one liquid phase, and phases (5) and (6) are only one phase, one being the denser version of the other. Nevertheless, we decided to split the liquid phase into two phases because the angular distribution functions and other properties are so different that we assume that the machine learning algorithms will also recognize this region as two phases.

Finally, there is a gas phase (7). In this phase, the molecules are mostly separated from each other, while there is also a small proportion of molecules that form hydrogen bonds with each other, which can also be seen in the angular distribution function.

#### 3.1.2 MB Parametrization: Machine Learning Determination

The phase diagram in Figure 3 was determined manually, i.e., we used various data (number of peaks in the angular distribution functions at different distances, changes in the diffusion coefficient, etc.) to manually determine where phase transitions are located. This procedure is quite time-consuming and tedious; therefore, we tried to find a more automatic approach using machine learning. As input data for the machine learning methods, we first used angular distribution functions at different radial distances. These distances are 0.7, 1.0, 1.73, 2.0, and 3.0. The distances were chosen on the basis of the fact that at these distances, peaks in the radial distribution function occur. These are therefore the distances at which the ordering of the molecules should be relatively strong and typical. Each distance also corresponds to a typical structure, e.g., 0.7 corresponds to two particles in LJ contact, 1.0 corresponds to a direct hydrogen bond between two particles, 1.73 corresponds to two particles bonded to the same third particle via hydrogen bonds, etc. If other distances were chosen that did not correspond to some typical structural patterns, it would be more difficult for the machine learning methods to distinguish between the phases, as the radial distributions would not be as structured. The angular distributions we used for machine learning were the same as in Figures 4 and 5, and each point in a distribution (or each point in figure) was treated as one dimension. Moreover, in order to lower the number of dimensions, we also reduced the resolution of the angular distribution functions so that we took only every third point in the function. To illustrate, instead of taking the angular distribution function values at angles −60, −59, −58, −57, ···, 60°; we only took every third point: −60, −57, −54, ···, 60°. In the end, each point in the pressure–temperature phase space had 125 dimensions containing information about the angular distribution functions. The dimensions were not normalized because we assumed that all angular distribution data were on the same scale. So the descriptors - angular distribution functions - are fed into unsupervised learning as they appear in the Figures 4 and 5; each (third) point in the figures is a dimension. There is no preprocessing at all, except that every third point is taken instead of all points (taking every third point reduces the computation time, but there is no loss of information, as the shape of the angular distribution functions remains the same). The angular distribution functions retain all the information they originally contained, including the translational and rotational symmetries, if they had any.

To concentrate the information about the phases of the rosewater model from the angular distribution functions to fewer dimensions, we used unsupervised machine learning techniques employed for dimensionality reduction. In all cases, the dimensionality of data was reduced to 3 dimensions. We reduced the dimensionality of the data to 3D for three reasons: First, because visualizing the data points in 3D is easy and thus allows for easier manual/visual evaluation of the results; second, because a larger number of dimensions increases the cost of the calculations. The third reason is that we wanted to keep the dimensionality of the data as low as possible without sacrificing the necessary information that would allow us to divide the data points into phases. Ideally, we would use only one dimension that reveals some kind of hidden property that describes the difference between phases. However, the efficient concentration of all necessary information on one dimension was not possible with the methods used in this work, so we used the least number of dimensions that gave relatively good results. The 3 dimensions are sufficient for our purpose, which is confirmed by very good results of some of the methods used here. There is a possibility that some of the methods could achieve better results if a larger number of dimensions were used. However, for reasons of consistency and comparison between methods, we used 3 dimensions for all methods. After dimensionality reduction, the points in the phase space were clustered by using clustering algorithms to group the phase points into possible phases. The methods used for dimensionality reduction are MDS, Isomap, Spectral Embedding, and t-SNE. We calculated the possible phases using combinations of all 4 dimensionality reduction methods and all 3 clustering algorithms, and then compared the results with the phase diagram that we had manually determined from the angular distributions.

Before we discuss the results of the individual method combinations in detail, let us look at the overall performance of the method combinations compared with the phase diagram, which was determined manually from the angular distributions. To quantitatively compare the results of the ML methods with the reference phase diagram, we simply compared all phase points where the phase diagrams were determined. If the diagrams had the same phase under the same conditions (in the same phase point), we would classify this as a match, but if the phases were different, this would be a disagreement, and finally, we simply calculated the fraction of matches across all compared phase points. In other words, the fraction of agreement is the ratio between the phase points with the correctly predicted phase and all of the phase points used in the calculation. In this way, we obtain the proportion of the ML-determined phase diagram that matches the reference diagram. In calculating this fraction, the two parts of the liquid phase were considered as one liquid phase, and the high-density solid phase at a pressure higher than 10.0 was also considered as one phase. In the Table 1 fraction of agreement between the phase diagrams calculated with each combination of methods and the reference phase diagram is given.

**Table 1:** Fraction of Agreement between the Phase Diagram of the Rose Model with MB Parametrization Calculated with Each Combination of Methods and the Reference Diagram^a^

dimensionality reduction	clustering algorithm	fraction of agreement
Isomap	K-means	0.934
Isomap	hierarchical	0.954
MDS	K-means	0.933
MDS	hierarchical	0.942
t-SNE	K-means	0.904
t-SNE	hierarchical	0.912
t-SNE	DBSCAN	0.734
spectral em.	K-means	0.866
spectral em.	hierarchical	0.727
spectral em.	DBSCAN	0.837

a The diagrams are derived from angular distribution data.

As can be seen in the table, Isomap hierarchical clustering is quantitatively the most successful combination of methods. Isomap and MDS have a fairly similar fraction of agreement, which is not unexpected, as the methods are similar. The dimensionality reduction method that stands out negatively is spectral embedding, as the agreement of its results with the reference diagram is relatively lower than the results of the other methods. Now that the success of the method combinations has been summarized quantitatively, we can examine the results of the different methods in more detail and qualitatively. Note that one method may provide a better quantitative match with the reference diagram while the other method provides a poorer quantitative but better qualitative agreement. This means that the first method may produce more phase points that are indexed to the same phases as in the reference diagram while also producing some unwanted/unexpected boundaries between phases. One such example is the MDS-hierarchical clustering and MDS-k-means clustering methods, where hierarchical clustering gives a quantitatively better agreement but k-means gives a qualitatively better agreement, as hierarchical clustering places part of the liquid phase between two solid phases at pressure 8.0, but in the reference diagram there is no liquid phase between these two solid phases.

What follows is a more detailed analysis of the results of the various methods, focusing more on the qualitative aspect of the results, that is, how sensibly the predicted phases are arranged in the phase diagram. The first dimensionality reduction method we used is MDS. In Figure S1, one can see the proposed phase diagrams obtained by combining MDS with hierarchical and k-means clustering. In addition to the phase diagrams, there are also 3D diagrams that show how the phase points are located in 3D space, where the dimensions are the output components of the MDS. Different colors indicate different phases. The colors in the phase diagram and the corresponding representation in the new 3D space are the same, so that the position of the points in the pressure–temperature diagram and the new component space can be compared. In Figure S1, the data were clustered using hierarchical clustering and k-means clustering. DBSCAN clustering was also performed, but the results were so much worse than the previous two methods that it was not included in the results. The MDS-DBSCAN combination does not provide good results because the data points are not adequately separated in the 3D space of the MDS output components, so that DBSCAN can be used. For DBSCAN to cluster the data points efficiently, the data points must be separated in such a way that the areas with high point density are separated by the areas with low point density. As can be seen from the figures, MDS (and also Isomap) places the data points relatively homogeneously in the new 3D space, while t-SNE places the points in the new 3D space in such a way that there are “islands” with high point density separated by empty space. For this reason, DBSCAN fails to cluster the data points projected into the new space with MDS or Isomap, while DBSCAN easily clusters the points when t-SNE is used to project the points into the new space. Comparing the phase diagrams obtained here with the phase diagram in Figure 3, it becomes clear that these two combinations of methods were very successful. Both combinations were able to find all of the phases that we had manually located. In addition, they split the high-pressure solid phase into two parts: a low-temperature part and a high-temperature part. The algorithms found that these parts of the high-pressure phase are so different from each other that they can be considered separate phases. The difference in the position of these high-pressure phases is also clearly visible in the projections onto the MDS output components, where the points from the low-temperature part of the phase are relatively scattered in space on the right side of the image. We also manually inspected the angular distribution functions and the snapshot in this region and concluded that it is likely to be a single phase and that the increased temperature reduces the order of the phase, and thus the peaks in the angular distribution functions are more merged and less pronounced. Both methods were also successful in determining the narrow belt of the liquid phase between the hexagonal solid phase and the gas phase. This demonstrates the superiority of machine learning over manual examination of the data. To find this narrow belt of the liquid manually, we had to look at the diffusion coefficient and fit it to the extrema of the thermodynamic functions. We were not able to find this belt of the liquid phase manually using only the angular distribution functions, while the machine learning methods found it effortlessly. Overall, both methods gave excellent results, but there are some differences in the way the methods cluster points in the same space. The phase diagram clustered with k-means is more similar to our reference phase diagram. This is mainly due to the prediction of the dense liquid phase, which agrees more with the reference diagram than the case of hierarchical clustering, where this phase also lies between two solid phases at a pressure of 9.0. Another point is that the pressure range of the same phase is better predicted by k-means. Overall, the differences between the phase diagrams obtained with the two clustering algorithms from points placed in the new space with MDS are relatively small. Therefore, it is also difficult to say why one method outperforms the other quantitatively, while the other is more successful qualitatively. In fact, both methods predict the same phases under the same conditions, while the boundaries between the phases are slightly different because the clusters are defined differently in the methods.

**Figure 6:**
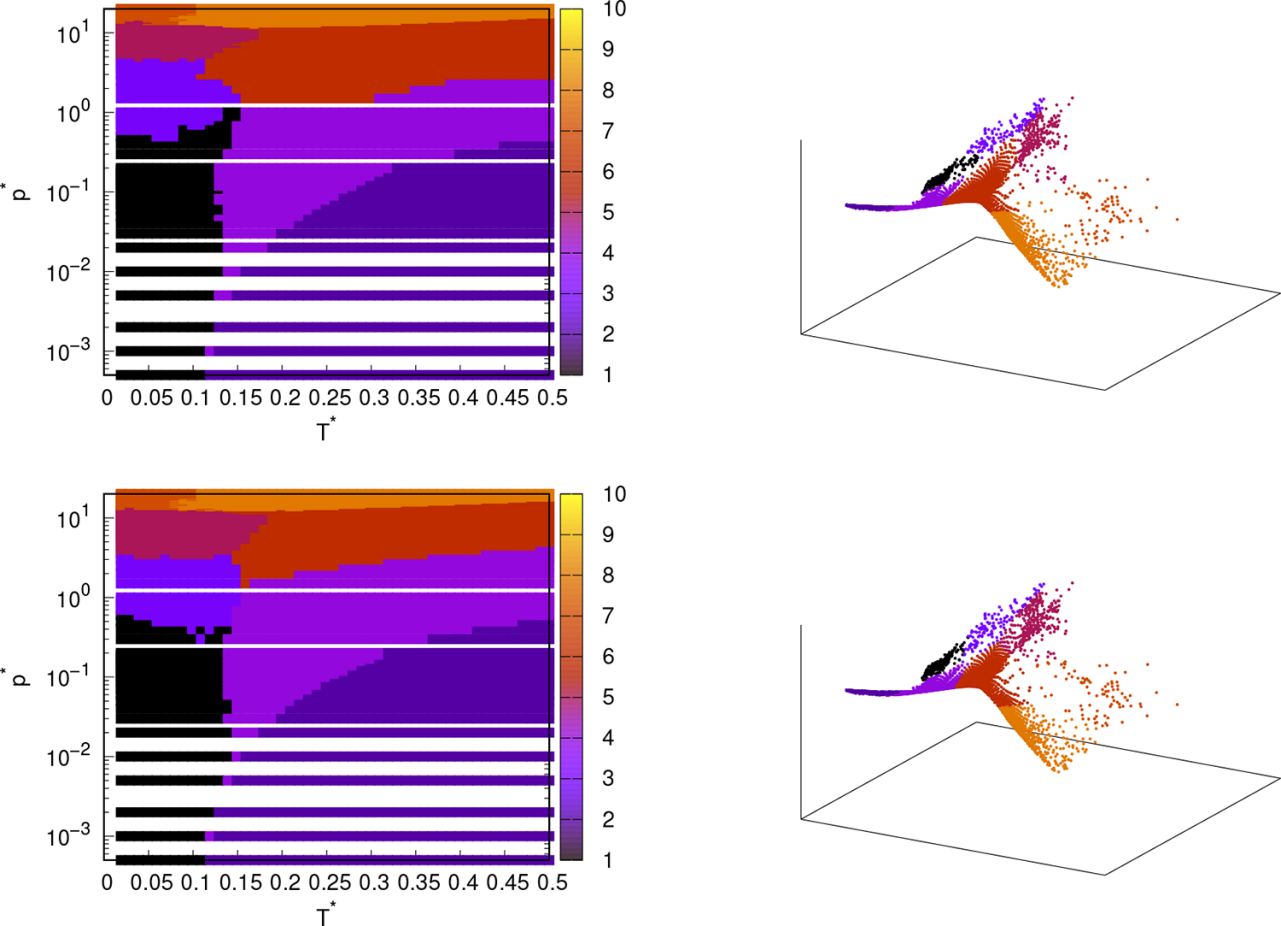
Pressure–temperature phase diagram of rose model with MB parametrization (left column) and projection of the same points on isomap output components (right column); obtained from angular distribution functions from MD simulations. Isomap was used to decrease the dimensionality of the data, and then clustering algorithms were used to cluster the data: hierarchical clustering (first line), k-means clustering (second line). DBSCAN clustering was also used, but the results were not useful. Only the phase points used in the calculations are displayed. For this reason, the phase diagrams have white areas where no calculations were performed.This applies to all phase diagrams shown in this paper.

Isomap also delivered very good results, which is expected as isomap is sort of an improved MDS. In Figure 6, phase diagrams and projections of data onto new components are shown. The clustering algorithms used in combination with Isomap to determine the phase diagram are again hierarchical clustering and k-means. Similar to MDS, DBSCAN was also unsuccessful with Isomap, as the results were much worse than the results of hierarchical and k-means clustering. Isomap requires the user to specify the number of neighbors for each point. In our case, this number was 100. The results are not very sensitive to the isomap parameter - number of neighbors. In this work, we used 100 for this parameter. Usually, this parameter is much smaller (e.g., 5–10), but in our case, it turned out that a very large value of this parameter leads to better results, probably because it better preserves the global structure of the data. However, even with a smaller value of the parameter, down to 30, the results were still very similar. With an even smaller number of neighbors (e.g., 5–10), however, the results became worse, as the global structure of the data was lost during the process. Comparing the phase diagrams obtained with Isomap and MDS, they are very similar. All 4 combinations of methods found the same phases, but the boundaries between the phases differed slightly from method to method. It was expected that the methods would give similar results, as MDS and isomap are similar methods. Essentially, both methods attempt to maintain the pairwise distances between data points when moving points from high to low-dimensional space. The main difference between isomap and MDS is that isomap uses geodesic distances between points on a manifold rather than just Euclidean distances. In Figure S1, there is a small region of different phases within a hexagonal phase near temperature 0.1 and pressure 0.03, but in Figure 6, there is no such area. This difference could be due to the differences in the dimensionality reduction methods. In the phase diagrams from MDS, these “islands” in the low-density hexagonal phase belong to a denser solid phase that exists at higher pressures than the low-density hexagonal phase. In the original high-dimensional space, the “island” points can be close to the denser solid phase if Euclidean distance is used as a metric, so these points will also be close to each other in the new low-dimensional space. However, if isomap uses the geodesic distance instead of the Euclidean distance, the “island” points will no longer be close to the points of the denser solid phase. This gives isomap a clear advantage over MDS. Both clustering algorithms in combination with Isomap also predict the narrow belt of the liquid phase between the solid and gaseous phase. The boundaries between the phases calculated with Isomap are slightly different from those of MDS. Isomap assigns a larger pressure range to the solid phase at pressure 10.0 than MDS, while MDS produces a phase at pressure 1.0 that covers a larger range than the one calculated with Isomap. In this sense, the diagrams of MDS match better with our reference diagram. However, Isomap is better at predicting the dense liquid phase as it matches better with that in the reference diagram. Most importantly, in the isomap-hierarchical clustering combination, the liquid phase is not between two solid phases like in the MDS-hierarchical clustering at high pressure. In the case of Isomap, the high-pressure solid phase is again divided into two parts at a pressure of 20.0, and the low-temperature part ends at a lower temperature than in the calculation with MDS. The boundaries between the phases are difficult to determine precisely, as the properties (in this case, the angular distributions) often change continuously and there are no sudden shifts or jumps that would clearly indicate a phase transition. Therefore, it is difficult to separate possible phases both manually and with machine learning algorithms. This is probably also the reason why DBSCAN was not successful, because DBSCAN distinguishes areas with high point density, which are separated from areas with low point density. The exception is the phase transition to the gas phase, where we observed a relatively sudden change in the angular distributions. Of 4 combinations of dimensionality reduction and clustering algorithms, isomap-hierarchical clustering and MDS-k-means were the most successful. The advantage of isomap-hierarchical clustering is that there are no “islands” of other phases in the phases. However, MDS-k-means agreed better with the reference diagram when it comes to the boundaries between solid phases. Overall, Isomap-hierarchical clustering seems to be the better choice due to its versatility as it is a nonlinear dimensionality reduction technique. Nevertheless, all 4 combinations of methods used delivered exceptionally good results compared to the difficulties and uncertainties we had when we manually determined the boundaries between the phases using angular distributions.

The next dimensionality reduction method that we used is spectral embedding. Figures of the phase diagrams obtained with this method can be found in the Supporting Information. Of all of the dimensionality reduction methods we used, this method gave the worst results. Spectral embedding was used in combination with hierarchical clustering, k-means, and DBSCAN. These combinations all gave similar results, as they could basically predict the same phases, but with some differences. The only phase that was correctly predicted is the gas phase and the hexagonal densely packed phase at a pressure of 20.0. The methods were not able to distinguish between the high-density liquid phase and the high-density solid phase at pressures from 5.0 to 10.0. Figures of these results can be found in the Supporting Information.

**Figure 7:**
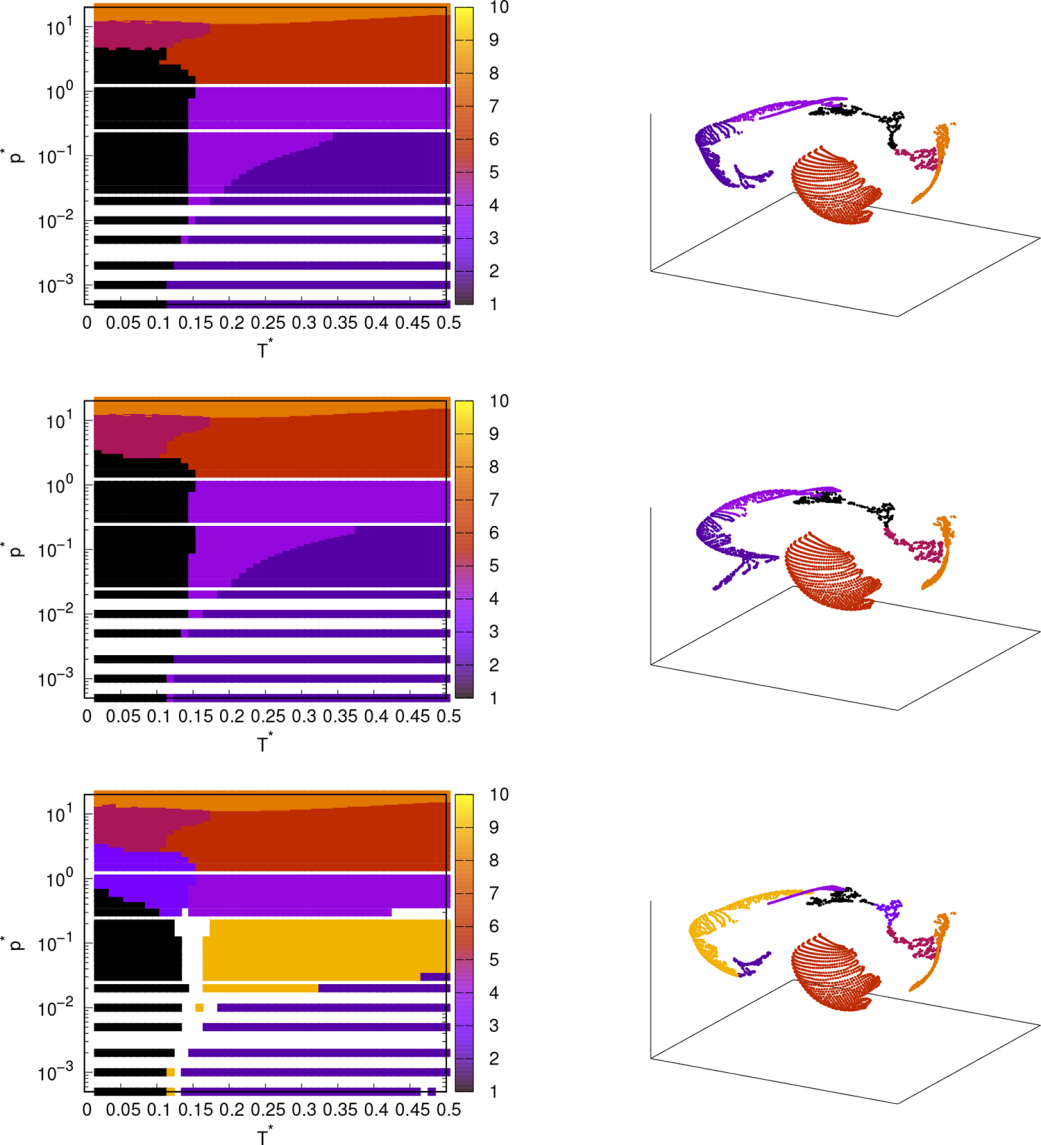
Pressure–temperature phase diagram of rose model with MB parametrization (left column) and projection of the same points on t-SNE output components (right column); obtained from angular distribution functions from MD simulations. T-SNE was used for dimensionality reduction, following by clustering algorithm: hierarchical clustering (first line), k-means clustering (second line) and DBSCAN (third line).

The last dimensionality reduction method used in this paper is t-SNE. The parameters for t-SNE are as follows: Perplexity was 20, early exaggeration was 12, learning rate was 100, and maximum iterations were set to 3000. In combination with clustering methods, t-SNE produced phase diagrams with good results (Figure 7), but not as good as the results of isomap or MDS. The combinations of t-SNE with hierarchical clustering and k-means yielded very similar phase diagrams, differing only slightly in some positions of the phase transitions. Both successfully predicted phase transitions between all phases in different states of matter. However, they find it difficult to distinguish between some solid phases. To be precise, the method determines phases (1) and (2) as one phase. The method distinguishes between phases (1) and (2) when we select a larger number of clusters into which the algorithm should divide the points. However, before the algorithm separates phases (1) and (2), it also separates some other phases that should not be separated according to our reference diagram. The combination t-SNE-DBSCAN is also quite successful. It successfully detects all solid phases, but the boundaries are at slightly different locations than those in the reference diagram. In addition, the method splits the liquid phase into 3 phases, so that all solid phases, except the high-pressure dense-packed one, have corresponding liquid phases with similar structure. There are some additional white spots in the phase diagram determined with DBSCAN. These are the points that were not assigned to a cluster, because they lie between the clusters with a high point density. From Figure 7, it can be seen that t-SNE is much more efficient in splitting points in the 3D space of the new component, because the points from different phases are separated much better than when using isomap or MDS. T-SNE focuses on the local structure of the data points rather than the global one, which works in our case, as we are looking for changes in the local structure that could indicate a phase transition. However, since the t-SNE does not preserve the global structure of the data well, the difference in the “strength” of the different phase transitions is not well preserved. For this reason, the algorithm splits some phases into many parts before finding all of the true phase transitions. For example, the algorithm started by splitting the liquid phase into several phases before splitting the solid phases (1) and (2) into two phases.

Of all of the dimensionality reduction methods presented so far, t-SNE is perhaps the most powerful. It also has many parameters and can therefore be fine-tuned to achieve the desired results. With more detailed fine-tuning, t-SNE in combination with the clustering algorithm might allow us to obtain an almost exact phase diagram. For our case, however, this is not optimal, because we want a method that can determine a phase diagram as automatically as possible from the data. This means that it should not require much additional knowledge of the system. We would also need the exact target phase diagram to fine-tune the method. But if we had such an accurate phase diagram, we could use supervised machine learning instead of unsupervised learning, which would probably be more accurate.

#### 3.1.3 Real Parametrization

The second parametrization of the rose model that we have used in this paper is the real parametrization. The main difference between the real and the MB parametrizations is that the MB parametrization has two characteristic radial distances: one for the LJ contact and one for the hydrogen bond. In the real parametrization, however, these distances are the same. One of the reasons for this difference is that the MB parametrization has some exaggerated volumetric properties that real physical water does not have. The real parametrization is therefore chosen so that these volumetric properties are not so exaggerated. This also indirectly makes the differences between the phases less clear, and therefore, the phases more difficult to locate.

The phase diagrams of the real parametrization were determined manually and by machine learning from the angular distributions in the same way as for the MB parametrization. The determination of the phase diagram was more difficult here, and the results were not as excellent as with the MB parametrization. Nevertheless, most main phase transitions were successfully determined both manually and automatically using machine learning methods. The results and their detailed discussion can be found in the Supporting Information.

### 3.2 Determining Phases from Thermodynamic, Dynamic, and Structural Data

Next, we tried to determine the phase diagram of the rose water model from various thermodynamic, dynamic, and structural quantities calculated during the MD simulation. The quantities we used as input data are enthalpy, density, isothermal compressibility, thermal expansion coefficient, heat capacity, diffusion coefficient, radial structure factor, average cosine of orientation of a molecule, average number of hydrogen bonds per molecule, fraction of molecules with zero, one, two, and three HB, structure factor for 3-fold symmetry, and structure factor for 6-fold symmetry. Each of these quantities represents one dimension. So the idea is to use a set of different (one-dimensional) data obtained by simulations and create a phase diagram from it. Some of the data may be correlated, some may contain similar information, and some may not. However, our goal is to use these data almost blindly, without manual selection and preprocessing (except for the standardization required in some dimensionality reduction algorithms), to obtain a phase diagram. In other words, we wanted to develop a method that almost automatically creates phase diagrams from different simulation results without worrying about which quantities contain the information needed to create the diagram. We used the following procedure. The quantities were first normalized (standardized to μ = 0, σ^2^ = 1), as some of the methods used require the dimensions to be normalized. Then the dimensionality was reduced from 15 to 3 dimensions using MDS, Isomap, spectral embedding, and t-SNE, and finally the data points were clustered using hierarchical clustering, k-means clustering, and DBSCAN.

#### 3.2.1 MB Parametrization: Automatic Approach

Let us first quantitatively examine the success of the methods used in generating phase diagrams from various simulation data. In Table 2, fractions of the agreement between the phase diagrams obtained with different methods and the reference diagram are shown. The calculation process was the same as in Table 1, with the exception that we used phase diagrams obtained from a combination of thermodynamic, dynamic, and structural data. Overall, the agreement of the phase diagrams obtained with all methods with the reference diagram is worse than when using angular distribution functions as input data for the methods. This was to be expected as the reference diagram is obtained from angular distribution functions and the ML input data were therefore previously directly linked to the reference diagram. On the other hand, different simulation quantities are now used as ML input data, which are theoretically more or less correlated with the angular distribution, since both the mentioned quantities and the angular distributions should differ from one phase to another. Here, the spectral embedding provided unexpectedly quantitatively good results, as the agreement scores were among the highest. However, the phase diagrams obtained with spectral embedding were qualitatively worse. Below is a more detailed qualitative analysis and discussion of the phase diagrams of the rose model with MB parametrization obtained from the combination of simulation quantities.

**Table 2:** Fraction of Agreement between the Phase Diagram of Rose Model with MB Parametrization Calculated with Each Combination of Methods and the Reference Diagram^a^

dimensionality reduction	clustering algorithm	fraction of agreement
isomap	K-means	0.731
Isomap	hierarchical	0.692
MDS	K-means	0.604
MDS	hierarchical	0.691
t-SNE	K-means	0.717
t-SNE	hierarchical	0.739
t-SNE	DBSCAN	0.491
spectral em.	K-means	0.756
spectral em.	hierarchical	0.751
spectral em.	DBSCAN	0.727

a The diagrams are derived from combination of thermodynamic, dynamic and structural data.

Starting with the phase diagram of the rose model with MB parametrization, Figure S11 shows the phase diagrams obtained with MDS and k-means/hierarchical clustering. Similar to the use of angular distributions as input data, DBSCAN in combination with MDS did not provide useful results. MDS provided quite good results in combination with k-means and hierarchical clustering. The methods were able to distinguish between gas, liquid, and solid phases, but the phase transitions are arranged somewhat differently than when calculating using angular distributions. In addition, the algorithm found only two solid phases in the low temperature range at different pressures, whereas three or even four phases were detected when using angular distributions. Both combinations of methods also split the gas phase into two parts, which is not the best solution. With MDS-hierarchical clustering, the liquid phase with high density could also be identified, but the same phase also reaches the high-pressure and low-temperature regions where the solid phase should be present. Both method combinations were able to find the high-pressure solid phase, but MDS-k-means determined the phase transition from the liquid to the high-pressure solid phase somewhat strangely, as the line between the phases decreases with temperature. When the two clustering algorithms in combination with MDS are compared, it is difficult to say which method is more successful, as neither of them produced a significantly better phase diagram. Unfortunately, none of the methods was able to localize the thin belt of the liquid phase between the solid and the gaseous phase. Projecting the data points onto the MDS output components shows that the points of some neighboring phases are close to each other, making it more difficult for the clustering algorithms to efficiently separate them into different phases.

In Figure S12, phase diagrams obtained by isomap and k-means or hierarchical clustering are shown. The results of the isomap are similar to the results of MDS, as the phases found by the isomap are basically the same as those found by MDS, except that the boundaries between them are slightly different. However, an obvious advantage of the phase diagrams obtained with the isomap is that the gas phase is not divided into two parts. Overall, the combination of the isomap-k-means methods seems to be the most successful, especially because the boundaries of the solid phase at a temperature of 0.05 and a pressure of 3.0 agree better with the phase diagrams obtained from the angular distributions. The isomap also predicts the upper part of the small belt of the liquid phase between the solid and gaseous phase. While MDS and isomap are similar methods, the combination isomap-k-means appears to be the best of the four combinations used here.

We also used spectral embedding for the dimensionality reduction of the data. However, the method, in combination with three clustering algorithms, yielded qualitatively worse results than MDS, isomap, and t-SNE as the spectral embedding determined a large part of the solid phase in a part of the phase diagram where the liquid phase should be present. The figures presenting phase diagrams obtained with spectral embedding can be found in the Supporting Information. The method combinations that included spectral embedding were able to find some important phase transitions: gas–liquid, liquid-high-pressure solid, and solid–gas. However, the methods also assigned quite a large part of the phase space around a temperature of 0.2 and a pressure of 1.0 to a solid phase, which we believe is a major problem.

**Figure 8:**
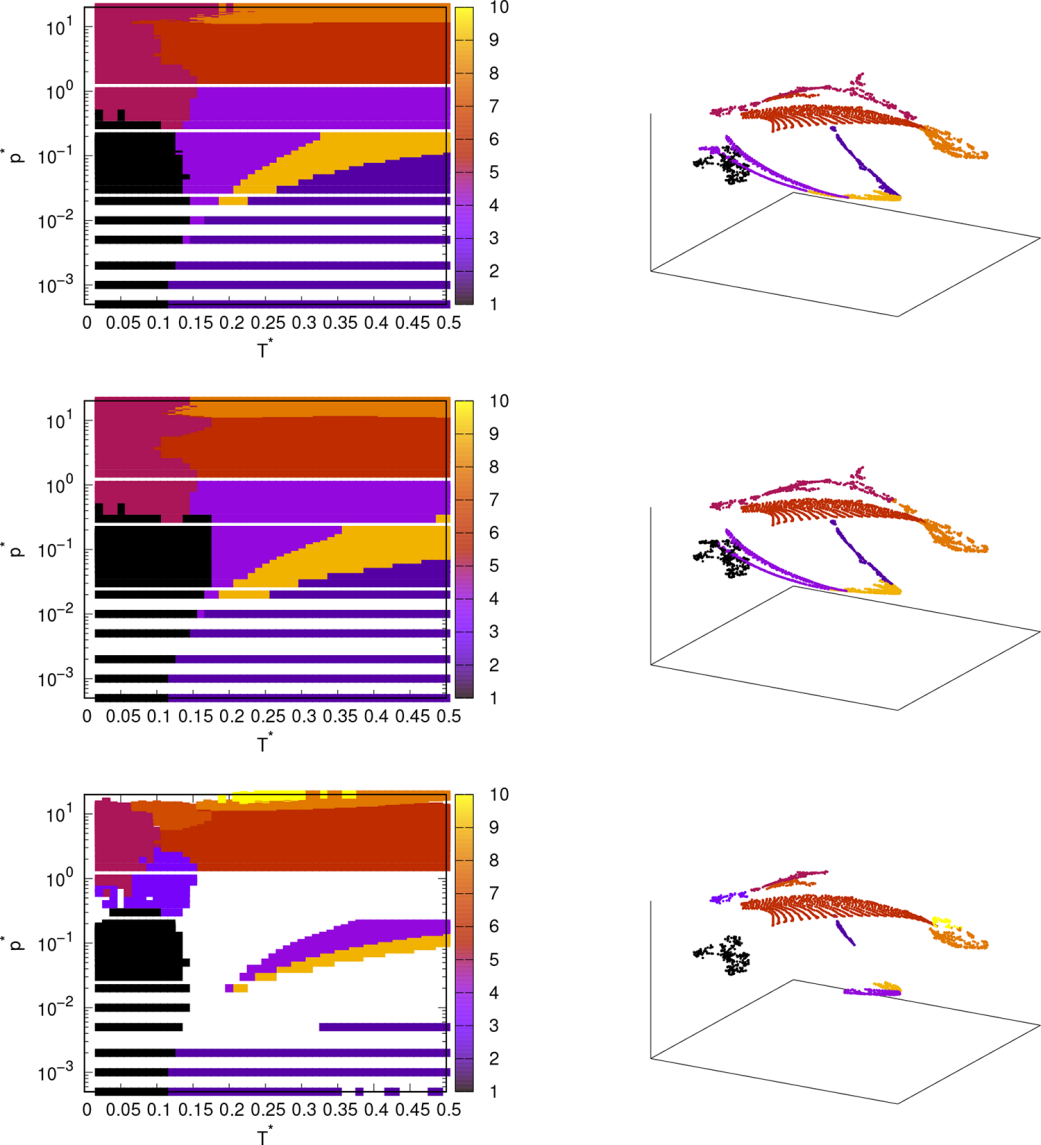
Pressure–temperature phase diagram of the rose model with MB parametrization (left column) and projection of the same points on t-SNE output components (right column); obtained from thermodynamic, structural, and dynamic data from MD simulations. T-SNE was used to decrease the dimensionality of the data, then clustering algorithms were used to cluster the data: hierarchical clustering (first line), k-means clustering (second line) and DBSCAN (third line).

The last method for dimensionality reduction is t-SNE (Figure 8). The parameters of the method are set to the same values as those used for angular distributions. This method was relatively successful, as it was able to predict most of the phases in the phase diagram, while the phase transitions also matched relatively well with those from the angular distribution functions. The combinations t-SNE-k-means and t-SNE-hierarchical clustering predicted quite similar phase diagrams, with the only significant difference being the boundaries of the different solid phases. Both methods also divided the gas phase into two parts, which is understandable since the second part falls in the supercritical fluid region. For the solid phases, k-means is more successful at higher pressures, as the boundaries of the solid phase below pressure 10.0 match better with the reference diagram, while hierarchical clustering is better at pressures from 0.01 to 1.0. On the other hand, DBSCAN gives different results. The problem with DBSCAN is the large number of data points that were not clustered. The two parameters in DBSCAN (minimal number of neighbors and maximal distance) were varied. We used 80 as the number of neighbors and varied the distance. With a high distance, there were no data points outside the clusters, but the number of clusters was low. If the distance was small enough, clustering was more successful, but many points remained uncategorized. The advantage of DBSCAN is that it succeeds in clustering solid phases at low temperatures better than the other two clustering algorithms. It is also interesting that when using t-SNE, the high-density liquid phase is so clearly separated from the other phases in the 3D projection onto the t-SNE output components. This separation can be seen when both angular distributions and thermodynamic data are used as input data. Of the methods using t-SNE (and also MDS and Isomap), the combination of t-SNE-hierarchical clustering is the most successful. The input data set used here is quite complex as it consists of many different quantities, some of which may be more important for separating the phases, while others may be less important because they contain less information. Therefore, it is more difficult for all methods to separate the phases on this basis than it was when angular distribution functions were used as input data. Here, the combination t-SNE-hierarchical clustering seems to be the most successful in predicting phase diagrams because, when t-SNE is used for dimensionality reduction, the reduction focuses more on preserving the local structure of the data as opposed to MDS and Isomap, where the global structure is more likely to be preserved. Therefore, t-SNE is more successful in finding differences between phases, as the differences between phases are easier to see locally than when looking at the global positions of the data points. The reason for the difficulty in using a more global structure is that some of the dimensions (quantities) may be relatively similar over a larger part of the phase diagram, thus reducing the differences between the positions of the points when viewed globally. Let us assume, for example, that we have density as a function of the temperature. At low temperature, the density is low, then it increases with increasing temperature up to a maximum density at moderate temperature, and then it decreases and reaches a low density again at high temperature. If we look at the global position of the points in the density dimension, the points at low and high temperatures are close to each other, so it would be difficult to separate them. On the other hand, if we look at the local structure, we see that the points at low and moderate temperatures can be separated, while the points at moderate and high temperatures can also be separated. Moreover, the distance between high and low temperature density points would not be preserved in the dimensionality reduction if we consider the local structure instead of the global structure, where the distance is preserved. After the dimensionality reduction, the clustering algorithm is applied. The differences in the success of the clustering algorithm depend on the arrangement of the data points in the new 3D space. Here, hierarchical clustering was more successful than k-means, as k-means is more successful due to the algorithm when the clusters are more uniformly shaped (more spherical), but here we have many long and thin clusters that are easier to cluster with hierarchical clustering, which uses the proximity between two points.

#### 3.2.2 MB Parametrization: Intelligent Approach

These approaches to calculating phase diagrams from thermodynamic, structural, and dynamic data were relatively automatic in the sense that we simply fed the data into the algorithm without considering which quantity was significant and which quantity contained which information. To get a better phase diagram from the simulation data, we also tried an approach that incorporates some knowledge about the system. One of the main differences between phases in the gaseous, liquid, and solid states is the value of the diffusion coefficient. Different phases have different structural, thermodynamic, and dynamic properties. Some properties can be similar in different phases. For example, the densities of two solid phases may be similar, while the ordering of molecules in these two phases is different. In the case of water, ice, liquid water, and water vapor clearly have different diffusion coefficients and can therefore be distinguished by them. We have only used diffusion here to distinguish between gaseous, liquid, and solid phases. Other properties were used to distinguish between solid phases (which have a similar diffusion coefficient). With this idea in mind, we first separated the phase space based on the diffusion coefficient. Previously, when we used the diffusion coefficient to create a reference diagram, we adjusted the limits of the diffusion coefficient to the extrema of the fluctuating thermodynamic functions (heat capacity, etc.). Here we tried to do this more automatically and therefore used sequences of DBSCAN. We started with diffusion data from points at all temperatures and pressures and performed DBSCAN on diffusion. This is because when diffusion is compared, the gas phase data points have greater diffusion, and the values of diffusion are also more scattered. On the other hand, the diffusion of the condensed phases is more concentrated around lower values. On the other hand, the data points of the condensed phase have a higher point density than the points of the gas phase. Therefore, DBSCAN can identify the condensed phase data points as clusters, while the gas phase points are outside the cluster. This process with DBSCAN was repeated three times, each time removing points from the phase with a lower diffusion point density. The number of core neighbor points was set to 60 in all cases, and the distance between neighbors was set as follows. The diffusion was first normalized. Then the plot of the distance to the k-th nearest neighbor versus data points sorted was constructed. The neighbor distance was then chosen at the value where the line on the graph falls almost to zero and the line becomes flat. In this way, we obtained the gas phase, two parts of the liquid phase, and a solid phase. To then distinguish between the different solid phases, we used a procedure similar to that before for the automatic approach. We used the phase points of the solid phases as input data, with each point having the same dimensions as before in the automated approach. The only variable excluded from the dimensions was the diffusion coefficient, as we already used it in DBSCAN. The dimensions were then standardized. For dimensionality reduction, we used MDS, Isomap, and t-SNE. After dimensionality reduction to 3 dimensions, clustering algorithms (k-means, hierarchical, DBSCAN) were used.

Table 3 shows fractions of agreement between the reference diagram and the phase diagrams obtained with different methods using the approach described above. It is obvious that the more intelligent use of methods and different simulation quantities improved the results of the fully automated approach. In addition, all methods used here have achieved better results than those in the previous section. The separation of gas, liquid, and solid phases is the same in all phase diagrams in this section, as they were separated using DBSCAN based on diffusion with DBSCAN. The contribution of the gaseous and liquid phases to the fraction of agreement is therefore the same for all methods. However, the solid phases were separated using different combinations of methods, so the separation of the different solid phases is responsible for the differences in fractions of agreement shown in Table 3. Of all the method combinations used here, t-SNE-DBSCAN is quantitatively the most successful in predicting the phase diagram of the rose model with MB parametrization. Moreover, as discussed in the following section, it was also qualitatively the most successful. In the following, the results of the different combinations of methods used are presented, and the evaluation of their qualitative agreement with the reference diagram is discussed in more detail.

**Table 3:** Fraction of Agreement between the Phase Diagram of the Rose Model with MB Parametrization Calculated with Each Combination of Methods and the Reference Diagram^a^

dimensionality reduction	clustering algorithm	fraction of agreement
isomap	K-means	0.762
isomap	hierarchical	0.785
MDS	K-means	0.787
MDS	hierarchical	0.798
t-SNE	K-means	0.793
t-SNE	hierarchical	0.794
t-SNE	DBSCAN	0.812

a The diagrams are derived from a combination of thermodynamic, dynamic, and structural data using the procedure described above. The gas, liquid, and solid phases are separated based on diffusion by DBSCAN, while different solid phases are separated using different combinations of methods.

**Figure 9:**
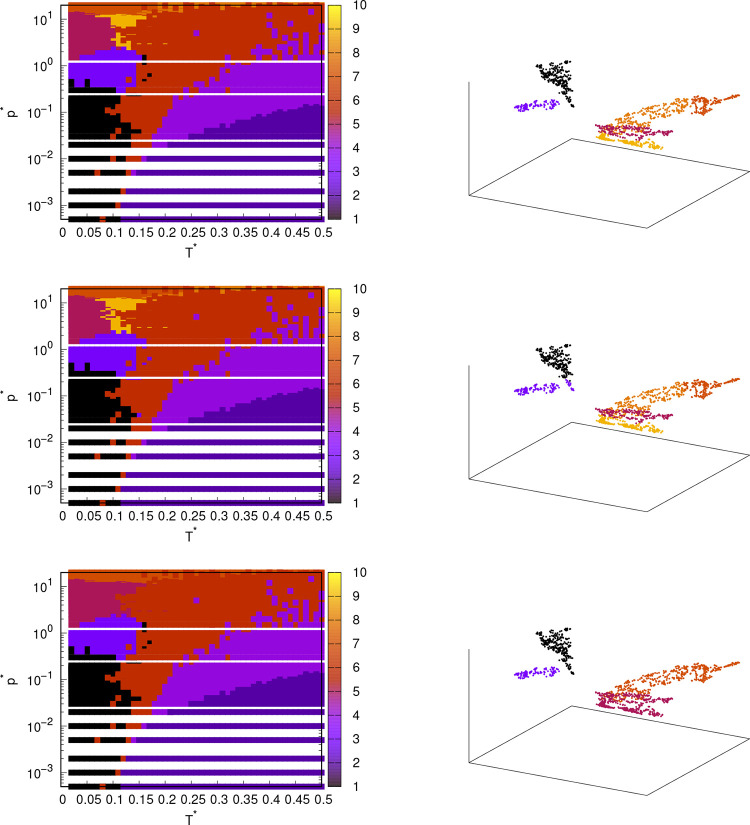
Pressure–temperature phase diagram of the rose model with MB parametrization (left column) and projection of the points of solid phases on t-SNE output components (right column); obtained from thermodynamic, structural, and dynamic data from MD simulations. First DBSCAN and diffusion coefficient were used to determine solid, liquid and gas phase, then T-SNE in combination with clustering algorithms (hierarchical clustering (first line), k-means clustering (second line) and DBSCAN (third line)) were used to separate solid phases.

In Figures 9, S14, and S15, phase diagrams of the rose water model with MB parametrization are shown; the diagrams were obtained using the method just described, where we used diffusion and other quantities separately to determine the diagrams. The first phase of the process, where DBSCAN and the diffusion coefficient are used to separate gas, liquid, and solid phases, is the same in all cases; therefore, the boundaries between these phases are identical in all following diagrams. The difference, however, lies in the division of the solid phases. The solid (combined), liquid, and gas phases shown in Figures 9, S14, and S15 are in excellent agreement with the phases in the reference diagram (Figure 3), which is to be expected as in both cases the diffusion data is used to determine these phase transitions. However, the results might differ because the diffusion data were treated with different methods. To construct the reference diagram, we fitted the diffusion values of the phases to the phase transitions obtained from the nested sampling. On the other hand, here we used DBSCAN without applying prior knowledge of the positions of the phase transitions and separated different states of matter based only on the point density of the diffusion data. The method we used basically just “peels” the data points layer by layer according to the increasing point density of diffusion. Moreover, the phase transitions determined by sequential DBSCANs of the diffusion are also more accurate than the phase transitions determined by clustering all quantities from the simulations, as shown in the previous section of the paper. One of the most important improvements is that a narrow belt of the liquid phase between the solid and gaseous phases has been successfully located here using DBSCAN. Note that the liquid phase is also split into two parts here, but the boundary between the two parts is different from the reference diagram, where angular distributions were used for splitting. In both cases, the additional liquid phase appeared near the phase transition to another phase. However, the partitioning of the liquid phase is different because it is based on different information. Nevertheless, in reality, there is only one liquid phase, and this liquid phase has slightly different properties in the different parts of the phase diagram.

Next, let us look at how well the dimensionality reduction methods combined with clustering algorithms separate the different solid phases. In this stage, all quantities obtained from the simulations were used as input data, with the exception of diffusion. Let us start with the most successful dimensionality reduction method, t-SNE. This method is the most effective in separating data points in the new 3D space. The parameters of t-SNE were the same as in the previous applications of t-SNE in this paper. In the right column of Figure 9, we see the projections of the solid state data points onto the new t-SNE components. The points are clearly divided into four clusters that are so well separated that we could cluster the points manually. When the clustering algorithms are used, the points are divided into different phases depending on the clustering algorithm used. The clustering algorithm that best separated the points is DBSCAN, which was expected since the points are perfectly separated in 3D t-SNE space, for using DBSCAN - high point density clusters separated by low point density. On the other hand, the success of DBSCAN was unexpected considering the previous applications of DBSCAN in this work, where DBSCAN was often the least successful clustering algorithm. The other two clustering algorithms separated points differently, but still quite well. The difference between DBSCAN and the other two clustering algorithms is that the two algorithms separated the phase at pressure 5 into two phases and the phase at pressure 20.0 into two phases, as well. The separation also makes sense based on the projection of the points into the new 3D space. This additional separation could correspond to differences in the properties. However, the question arises as to whether these are really separate phases or just one and the same phase with slightly different properties. The k-means and hierarchical clustering clustered the points almost identically. The only difference between the two is a small tail of points in the upper left cluster in the 2D projection. This tail corresponds to the few lonely points around pressure 0.5, which should be in a liquid state based on their position in the temperature–pressure diagram. All in all, the combination of t-SNE and DBSCAN seems to be the most trustworthy and can be quite powerful for clustering the data points if the input data is intelligently preselected and not just blindly thrown into the algorithm.

Next, in Figure S14, phase diagrams created with MDS and k-means/hierarchical clustering are shown. DBSCAN did not perform well in combination with MDS, so its results are not shown and are not discussed. The two phase diagrams in Figure S14 are very similar. They both predicted the same number of phases under the same conditions; they only differ in the position of the boundaries between the phases, although these boundaries are also similar. The phase diagrams obtained with MDS are also similar to those obtained with t-SNE and k-means/hierarchical clustering. All four phase diagrams contain all of the main phases, while the phase at pressure 5.0 is similarly divided into two parts. The difference between the t-SNE-k-means/hierarchical clustering and the MDS-k-means/hierarchical clustering lies in the division of the solid phases at high pressure. When using MDS, the low-temperature part of the high-pressure solid phase ends at a lower temperature than when using t-SNE. In addition, the MDS also predicts another phase in a small region at the lowest possible temperature. The reason for this additional small phase at low temperature and high pressure is probably the heat capacity and thermal expansion coefficient, which have significantly higher values in this part of the phase diagram than in any other part of the phase diagram.

Figure S15 shows phase diagrams obtained by combinations of isomap and clustering algorithms. The phase diagrams obtained here are similar to those obtained with MDS. All four combinations predict the same phases with slightly different boundaries. The results of isomap are worse compared to MDS if we look at the agreement with the reference diagram. The main problem with isomap’s diagrams is that the (additional) solid phase at pressure 5.0 and temperature 0.1 extends up to a pressure of 20.0; moreover, the solid phase at pressure 5.0 and lower temperatures has no boundary to the liquid phase. If the number of phases in the algorithm is reduced, then other valid phase transitions disappear before the additional ones disappear.

Overall, the combination of t-SNE and DBSCAN seems to be best suited to determine the phase diagram from different simulation data, mainly because t-SNE separates the points very well so that they can be clustered unambiguously by DBSCAN. Furthermore, this combination of methods provides the most balanced results, which means that the methods find practically all expected phase transitions before some additional ones appear. The reason why this combination of methods leads to the best results is 2-fold, because each step has its own reason. First, t-SNE in this case reduces dimensionality more effectively than does MDS or Isomap. The reason for this is similar to the previous section, where we automatically determined phase diagrams from different simulation data. Here we have basically the same data as in the previous section, except that the gaseous and liquid phases have already been excluded from the data. This exclusion narrows the range of thermodynamic properties so that the methods can more easily distinguish between small differences between solid phases. This exclusion improved the effectiveness of all combinations of the methods used. The reason that combinations with t-SNE are generally the most successful here is that they focus on preserving the local data structure rather than the global data structure, as the solid phases are easier to distinguish based on local differences between phase points, while the differences at the global level may not be as pronounced. For the second method in combination, the clustering algorithm, DBSCAN, is the most successful. The reason why this algorithm is most successful here is simple: the placement of the data points in the new 3D space is perfect for this, as the points are placed in high point density clusters separated by low point density spaces. It seems that the t-SNE-DBSCAN combination is generally very good, as t-SNE places the points in such a way that DBSCAN can separate them perfectly. However, if we go back to the previous section and feed all the data into the algorithm (without first separating the gas and liquid phases from the solid phase), t-SNE-DBSCAN was not quantitatively the most successful. The separation of gas and liquid phases shown in Figure 8 was quite poor, but the separation of the solid phases was very good. So we came up with the idea of first separating the solid phases from the liquid and gaseous phases, which we did in this section. The main point here is that blind automation does not give the best results, but if the data are selected more intelligently, the results of the methods can improve significantly.

#### 3.2.3 Real Parametrization

The phase diagram of the rose model with real parametrization was determined from simulation data in the same way as for the MB parametrization. The figures with the phase diagrams of the real parametrization obtained with different methods can be found in the Supporting Information, together with a more detailed discussion of these results. First, we tried to determine it directly from all of the simulation quantities in the entire phase space. Similar to the MB parametrization, the results were mixed, and the agreement with the reference diagram and the phase diagrams from the angular distribution functions was inconsistent. Some of the most important phase transitions were successfully predicted, while others were not. Furthermore, in many cases, additional phase transitions that probably do not exist were predicted by the algorithms. One of the reasons that an automatic approach, where we simply take all the data into the algorithm, is not optimal is that the differences between the different phases vary in size. For example, the difference between the solid and the gaseous phase is large, while the difference between two neighboring solid phases is small. Therefore, if both phase transitions are determined in the same step, we may miss the transition between the solid phase because the two solid phases are relatively similar to each other compared to the gaseous phase. To separate the solid phases more efficiently, it is therefore advisable to separate the more “drastic” phase transitions and the “mild” phase transitions.

For the rose model with MB parametrization, the approach of first separating the solid, liquid, and gaseous phases based on the diffusion coefficient and then further subdividing the solid phases based on other simulation data showed a significant improvement compared to processing all quantities from the simulations in one step. The same procedure was also used for the model with real parametrization. The separation of solid, liquid, and gaseous phases based on the diffusion coefficient using DBSCANs was successful and in good agreement with the reference diagram. On the other hand, the separation of the different solid phases of the rose model with real parametrization was not as good as with the MB parametrization. The reason for the more difficult separation of the solid phases is that the solid phases of real parametrization are less diverse than those of MB parametrization. Therefore, the differences in the properties of the solid phases are less pronounced. Another possibility is that the real parametrization of the model has only one solid phase and that the methods used to find different phases have merely split the one phase into parts with different properties.

## 4 Conclusions

In this work, unsupervised machine learning was used to determine the phase diagram of the rose water model. The Rose water model is a simple two-dimensional water model in which hydrogen bonds are explicitly modeled. Two parametrizations of the model were used in this work, namely, the MB parametrization and the real parametrization. The MB parametrization provides more diverse properties under different conditions, while the real parametrization has some properties that are more similar to those of real physical water. We focused more on the MB parametrization because it has a more diverse phase behavior, while we also determined the phase diagram of the real parametrization of the model. Initially, the reference diagrams were created manually from different simulation data. The purpose of these diagrams was to serve as a reference against which the machine learning results could be compared. The basic structure of the manually determined phases was then presented with angular distributions and snapshots of the system. To determine a phase diagram in a more automatic way, we used unsupervised machine learning methods. We used combinations of different dimensionality reduction methods, together with clustering algorithms. Two different data sets were used as input data. The first was angular distribution functions of water molecules at different radial distances, while the second data set contained different thermodynamic, structural, and dynamic quantities calculated during MD simulations. When using angular distribution functions as input data, the combination of methods that yielded the most accurate phase diagram was isomap-hierarchical clustering, which enabled virtually automatic determination of the phase diagram from angular distributions. Another promising combination is t-SNE-DBSCAN, which has some drawbacks but provides excellent separation of the different phases in a low-dimensional space. When using thermodynamic, dynamic, and structural data as input for machine learning, we found that the approach that gives the best result consists of two parts. First, we separated the solid, liquid, and gaseous phases based on the diffusion coefficient. Here we used a sequence of DBSCANs, and the results were excellent. In the second part, we separated the solid phases. This was done by combinations of dimensionality reduction methods and clustering algorithms, while the input data consisted of all of the previously mentioned simulation quantities except diffusion. The combination of methods that gave the best result here was t-SNE-DBSCAN, while many other combinations also gave excellent results. The phase diagrams obtained from the two data sets showed excellent semiquantitative agreement. In total, 4 different solid phases were found as well as a gas phase and a liquid phase. In the case of the real parametrization of the rose model, the results were less good, as the phases are more similar to each other and therefore more difficult to separate. The separation into solid, liquid, and gaseous phases was very good, while the division into different solid phases was less successful. A possible reason for this could be that the real parametrization actually has only one solid phase and that our method in this case splits the phase into parts with different properties. Overall, there is no combination of dimensionality reduction method and clustering algorithm that is superior to all others, regardless of the data set to which it is applied. The structure of the input data set and the distribution of the information in this data set significantly influence the success of the methods. Therefore, the method should be selected based on the structure of the input data used. However, some combinations of methods work better together. For example, t-SNE places the data points in well-separated clusters with nonuniform shape, so DBSCAN is a good clustering algorithm for the combination. Isomap, on the other hand, arranges the data points in a more continuous and uniform manner, making k-means a better method for combining. To summarize, the unsupervised machine learning approaches applied to the simulation data of the rose water model successfully generated a phase diagram of the rose water model. The presented approach requires minimal prior knowledge about the system under study and could therefore be applied to other systems (similar to or maybe even more complicated) to obtain their phase diagram.

## Supporting Information Available

Phase diagrams of rose model with MB parametrization obtained from angular distribution functions by MDS and spectral embedding; manually determined phase diagram of rose model with real parametrization, snapshots and figures of angular distribution functions typical for different phases of the model with real parametrization; phase diagrams of rose model with real parametrization obtained from angular distribution functions by all combinations of machine learning methods that were used in the study; phase diagrams of rose model with MB parametrization obtained from combination of thermodynamic, dynamic and structural quantities using MDS, isomap and spectral embedding; phase diagrams of rose model with MB parametrization obtained from combination of thermodynamic, dynamic and structural quantities using DBSCAN to identify gas and liquid phase and combinations of machine learning methods that include MDS, isomap and spectral embedding to identify solid phases; phase diagrams of rose model with real parametrization obtained from combination of thermodynamic, dynamic and structural quantities using DBSCAN to identify gas and liquid phase and all combinations of machine learning methods that were used in the study to identify solid phases; tables showing fraction of agreement between the reference diagram of rose model with real parametrization and different diagrams obtained with ML; and brief discussion of figures and tables (PDF)
