# Supplementary material for: Calculating a Phase Diagram of a Simple Water Model Using Unsupervised Machine Learning on Simulation Data
Source: J Chem Theory Comput. 2025 Apr 14;21(8):3867–87. doi: 10.1021/acs.jctc.4c01456 (PMC12020001; doi:10.1021/acs.jctc.4c01456)
Supplement: Supplementary file 1 — ct4c01456_si_001.pdf [file ct4c01456_si_001.pdf]

Calculating a Phase Diagram of a Simple Water Model Using Unsupervised Machine Learning on Simulation Data. SUPPORTING INFORMATION

Peter Ogrin and Tomaz Urbic*

Faculty of Chemistry and Chemical Technology,

University of Ljubljana, Vecna Pot 113, SI-1000 Ljubljana, Slovenia

(Dated: March 26, 2025)

* tomaz.urbic@fkkt.uni-lj.si

S1. SUPPLEMENTARY FIGURES AND RESULTS

A. Determining phases from angular distribution functions

1. MB parametrisation - Machine learning determination

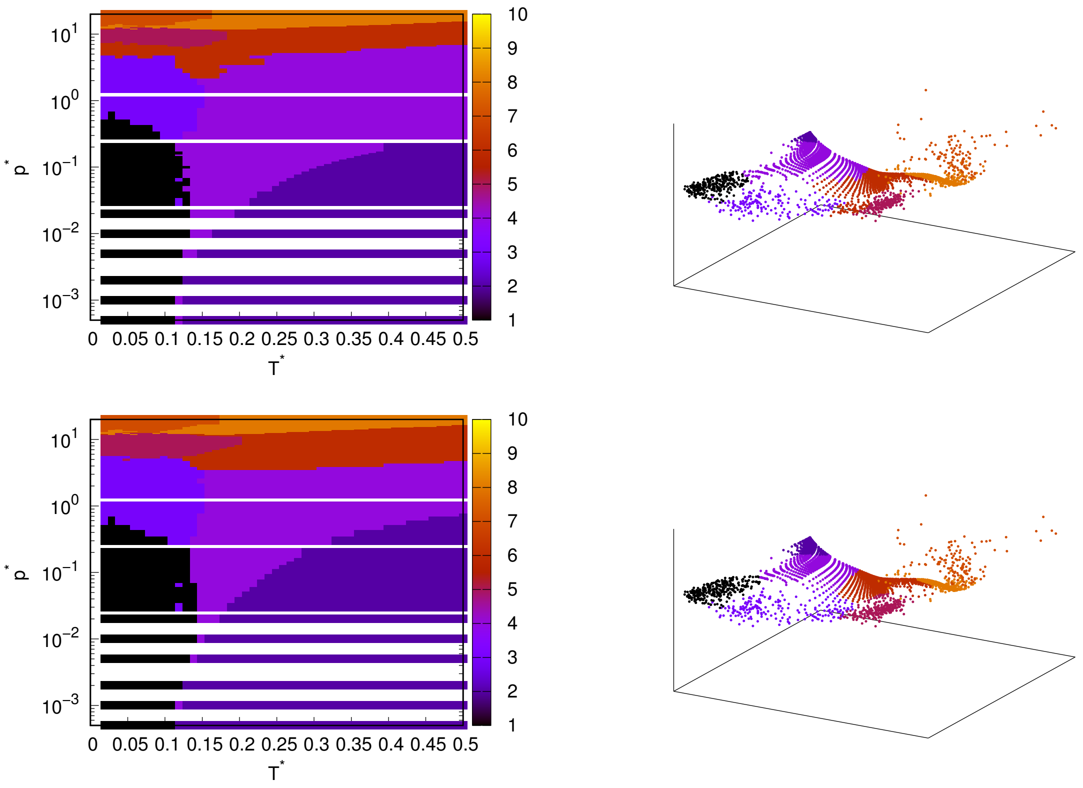

Figure S1: Pressure-temperature phase diagram of rose model with MB parametrisation (left column) and projection of the same points on MDS output components (right column); obtained from angular distribution functions from MD simulations. MDS was used to decrease the dimensionality of the data, then clustering algorithms were used to cluster the data: hierarchical clustering (first line), k-means clustering (second line). DBSCAN clustering was also used but the results were not useful. Only the phase points used in the calculations are displayed. For this reason, the phase diagrams have white areas where no calculations were performed.This applies to all phase diagrams shown in this paper.

In Fig. S2 phase diagram of rose model with MB parameterization. The digram was

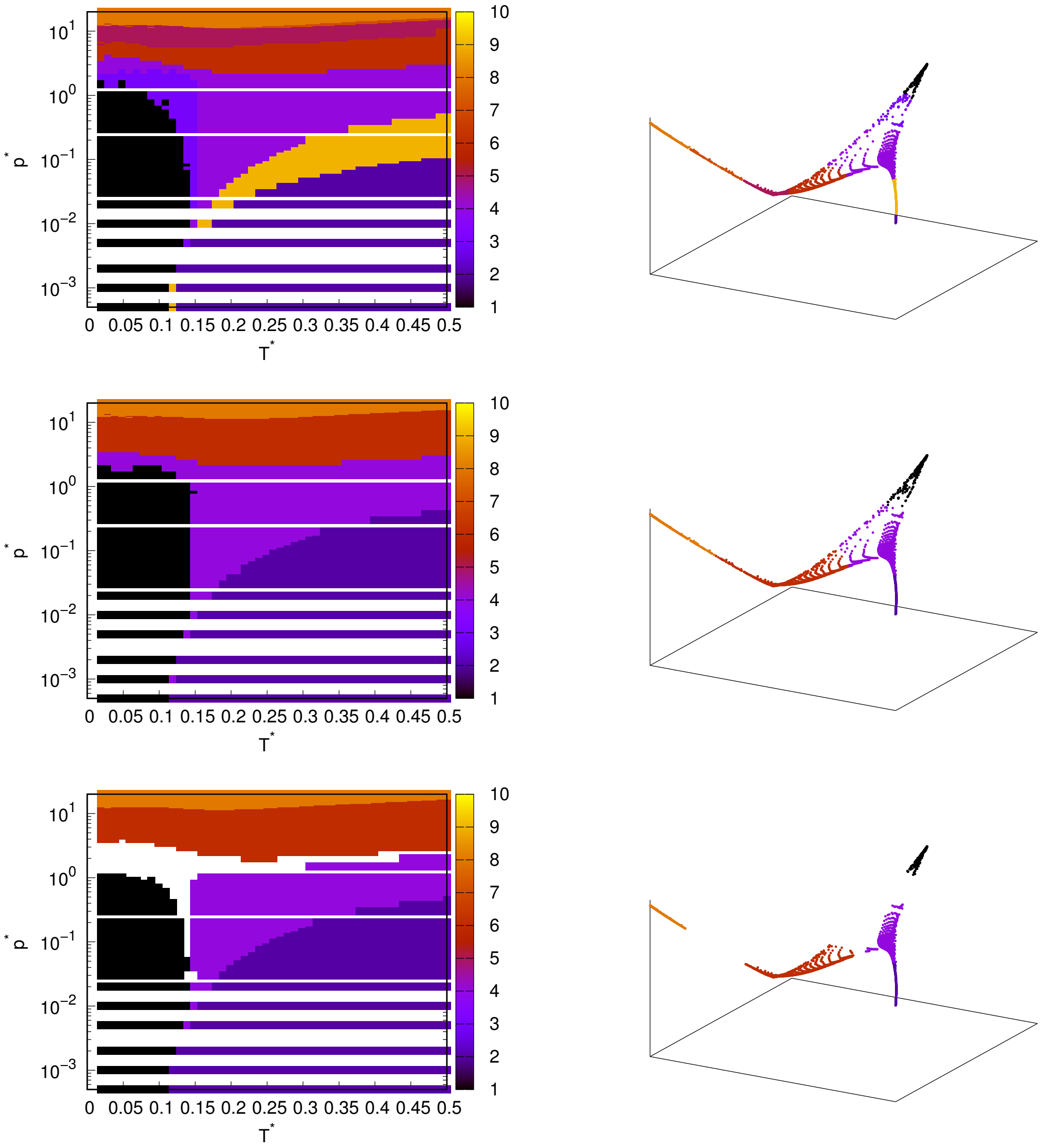

Figure S2: Pressure-temperature phase diagram of rose model with MB parametrisation (left column) and projection of the same points on spectral embedding output components (right column); obtained from angular distribution functions from MD simulations. Spectral embedding was used to decrease the dimensionality of the data, then clustering algorithms were used to cluster the data: hierarchical clustering (first line), k-means clustering (second line) and DBSCAN (third line).

created with spectral embedding and various clustering algorithms from angular distribution functions. Of all the dimensionality reduction methods used in this work, spectral embedding

gave the worst results. Nevertheless, spectral embedding in combination with clustering algorithms was able to predict some phase transitions, namely liquid-gas and liquid-solid phase transitions. However, the position of the liquid-solid phase transition differs slightly from the predictions of other methods. In addition, the method was also able to predict the location of the high-density solid phase. All clustering algorithms provide similar results here. While k-means and DBSCAN predict 5 phases, hierarchical clustering predicts 9 phases as some of the phases are split into multiple phases.

2. Real parametrisation - Manual determination

The second parameterization of rose model that we have used in this paper is the real parameterization. The main difference between the real and the MB parameterizations is that the MB parameterization has two characteristic radial distances, one for the LJ contact and one for the hydrogen bond. In the real parameterization, however, these distances are the same. One of the reasons for this difference is that the MB parameterization has some exaggerated volumetric properties that the real physical water does not have. The real parameterization is therefore chosen so that these volumetric properties are not so exaggerated. This also indirectly makes the differences between the phases less clear and therefore the phases more difficult to locate.

In Fig. S3 proposed reference diagram of rose water model with real parametrisation is shown. The diagram is calculated using the same procedure as the diagram of MB parametrization. Here too, the yellow shades represent solid phases, the blue shades represent liquid phases and the purple shades represent gaseous phase. The manual determination of this phase diagram was more difficult than the phase diagram of rose model with MB parameterization, as the differences between the phases are less clear. The diagram is mainly used for orientation when assessing the success of machine learning methods in determining the phase diagram. The angular distribution functions of the model with real parameterization are much less diverse than those of the model with MB parameterization. Therefore, it is more difficult to determine the phase transitions of the model with real parameterization based on the angular distribution functions.

In Fig. S4 and S5 angular distribution functions at different distances along with snapshot of the system are shown for each of the proposed phases from the phase diagram in Fig. S3.

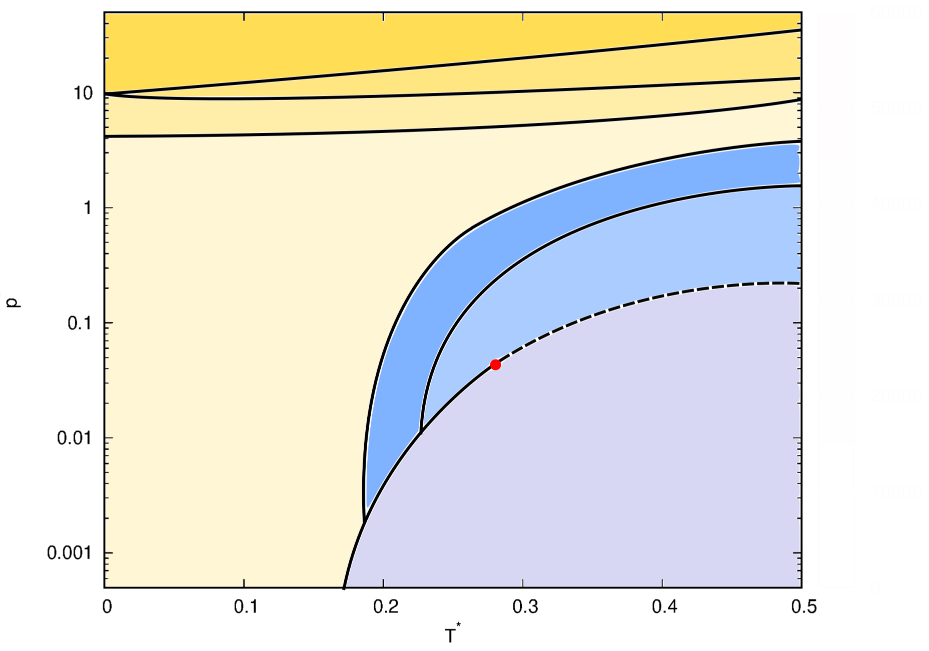

Figure S3: Pressure-temperature phase diagram of rose model with real parametrisation obtained from angular distribution functions by counting the number of the peaks in the functions. The diffusion data from simulation helped us to locate the liquid phases.

One can immediately see that these angular distribution functions and snapshots are less diverse than those of the MB parameterization. Let us start with phase (1), a solid phase in which a majority of the molecules are bound by hydrogen bonds to form a hexagonal network of hydrogen bonds. This phase is essentially the same phase as phase (1) of rose model with MB parameterization, but it is less ordered and further away from the ideal hexagonal lattice. The decrease in order is due to a characteristic radial distance that the real parameterization has instead of two distances in the MB parameterization. This, in combination with the "half-bonds" that can be formed by rose model, leads to a denser and less ordered structure.

Phase (2) is the liquid version of phase (1). In this phase, many molecules are still connected to HB, and some hexagons can also be found in the snapshots. The angular distribution functions of phase (2) are almost the same as those of phase (1), except that the peaks are less pronounced. Phase (3) is also a liquid phase. Here we divide the liquid phase into two parts - phase (2) and (3), because the radial distribution functions are so different that the machine learning algorithms could divide the liquid phase into two phases.

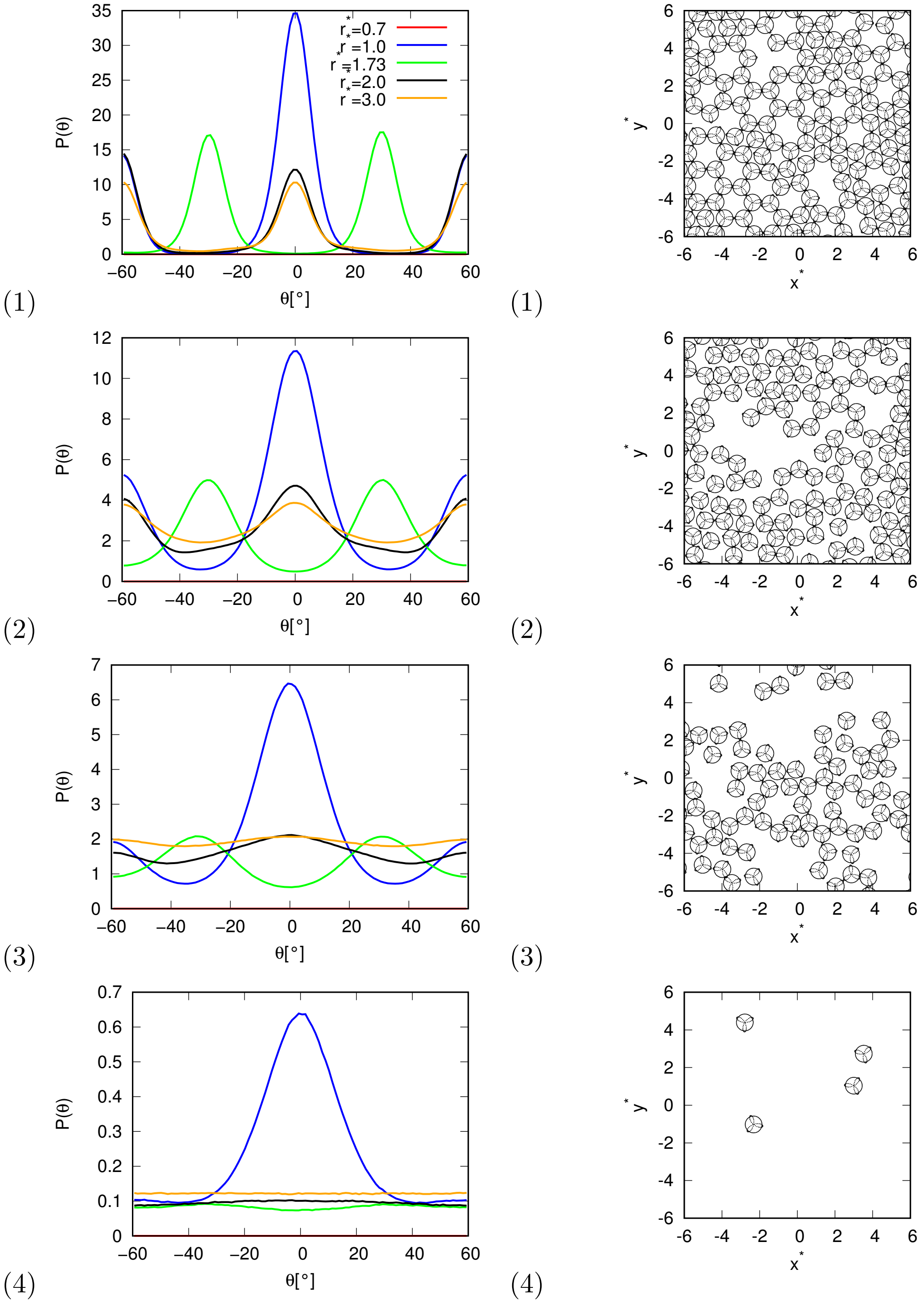

Figure S4: Angular distribution function between HB arm and line connecting centres of molecules at selected distance (left column), and snapshots of the system (right column). Distributions and snapshots are shown for different phases at conditions: (1) $p^* = 0.01$, $T^* = 0.06$, (2) $p^* = 0.02$, $T^* = 0.19$, (3) $p^* = 0.02$, $T^* = 0.23$, (4) $p^* = 0.01$, $T^* = 0.29$.

However, the structure of the two liquid phases is very similar. The difference is that phase

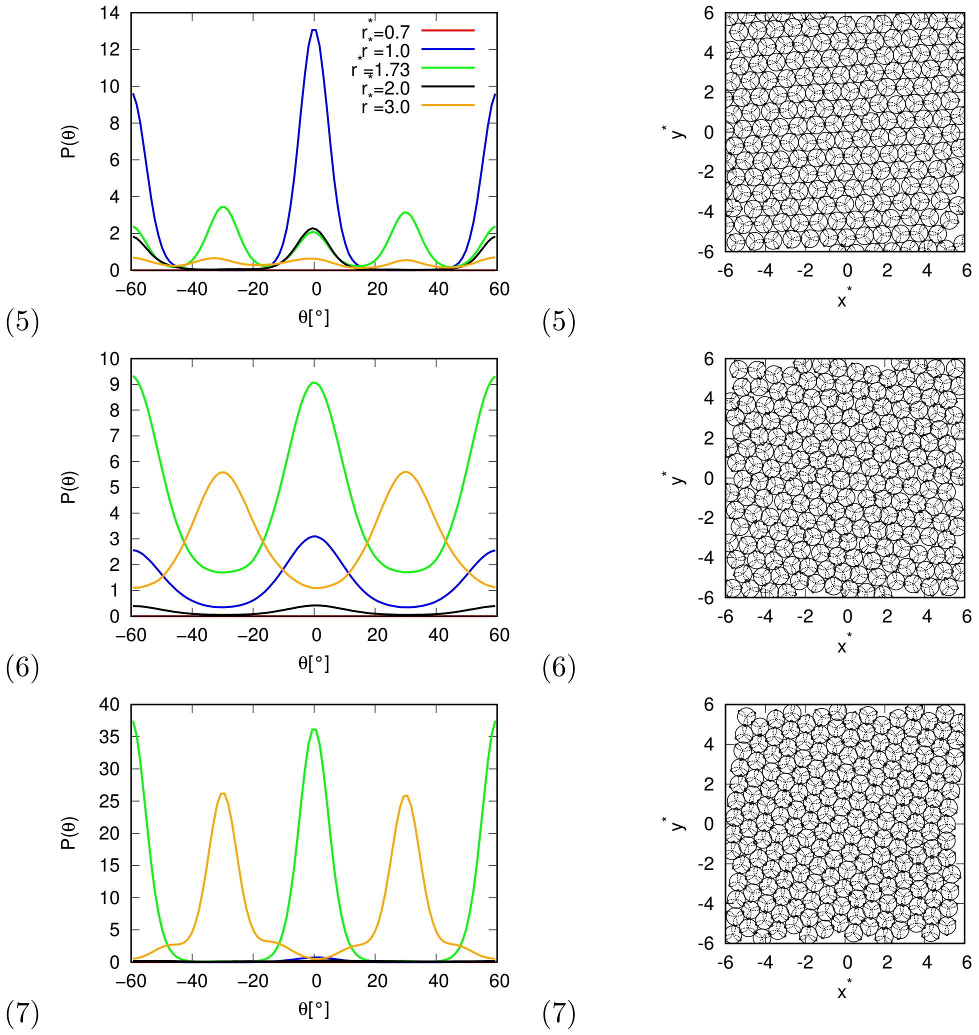

Figure S5: Angular distribution function between HB arm and line connecting centres of molecules at selected distance (left column), and snapshots of the system (right column). Distributions and snapshots are shown for different phases at conditions: (5) $p^* = 6.0$, $T^* = 0.09$, (6) $p^* = 15.0$, $T^* = 0.40$, (7) $p^* = 20.0$, $T^* = 0.10$.

(2) is denser than phase (3) and that phase (2) has more distinct peaks in the angular distribution functions than phase (3).

Of course, we also have a gas phase of the Rose model with real parameterization, which is phase (4). Similar to the MB parameterization, there is almost no structuring of the molecules into larger structures, however, there are still some molecules that have hydrogen bonds, which can be seen in the angular distribution function.

Then there are 3 solid phases at high pressure. The snapshots of all three phases are

similar, as all three phases are basically hexagonal dense packings, while there are some differences between them, e.g. the molecules in phase (5) are more aligned in straight rows than in phase (6). The differences between the phases can also be clearly seen in the angular distribution functions.

3. Real parametrisation - Machine learning determination

The manual determination of the phase diagram of rose water model with real parameterization was more difficult than with the MB parameterization, mainly because the differences between the phases are smaller due to less divers interactions. One would expect that machine learning algorithms could be more successful in determining phase diagrams in such cases because they could find some significant differences between phases that we missed in the manual determination. However, a prerequisite for the success of algorithms is that the input data contains the information that can be used to distinguish between different phases. The phase diagram of rose water model with real parameterization was determined using the same procedure as that of the rose model with MB parameterization. Angular distribution functions were used as input data and the same combinations of machine learning techniques were tried for the real parameterization as for the MB parameterization.

Table S1 shows fractions of agreement between phase diagrams determined by ML methods and reference phase diagram. The input data for ML methods are angular distribution functions. Quantitatively the most successful method here is combination isomap-k-means. The procedure used to calculate the fraction of agreement was the same as for MB parametrization of the model. The two parts of liquid phase were considered as one phase, moreover phases that are indexed as 1 and 3 in following diagrams were both considered correct predictions for the main solid phase.

When determining the phase diagram of rose model with MB parameterization, the two most successful dimensionality reduction methods were MDS and Isomap. In Fig. S6 pressure-temperature phase diagram of rose water model with real parametrisation is shown. The diagram was obtained from the angular distribution functions by MDS in combination with hierarchical clustering and k-means. The phase diagrams of the model with real parameterization obtained with MDS are quite similar to the reference diagram that we created manually, but there are some differences between them. Overall, the agreement

Dimensionality reduction	Clustering algorithm	Fraction of agreement
Isomap	K-means	0.891
Isomap	Hierarchical	0.806
MDS	K-means	0.688
MDS	Hierarchical	0.702
t-SNE	K-means	0.892
t-SNE	Hierarchical	0.883
t-SNE	DBSCAN	0.829
Spectral Em.	K-means	0.747
Spectral Em.	Hierarchical	0.806
Spectral Em.	DBSCAN	0.614

Table S1: Fraction of agreement between the phase diagram of rose model with real parametrisation calculated with each combination of methods and the reference diagram. The diagrams are derived from angular distribution data.

between the diagrams calculated with MDS and the reference diagram is better for the MB parameterization than for the real parameterization. However, MDS is still quite successful in combination with clustering methods. Both hierarchical clustering and k-means successfully predict the gas phase and the liquid phase, and the transition to high-pressure solid phases is predicted under the right conditions. On the other hand, the high-pressure solid phases calculated with MDS differ from those predicted manually by counting the peaks in the angular distributions. For example, in the high pressure range from pressure 5.0 to 10.0, we found only one phase by counting the peaks in the angular distributions. However, MDS in combination with clustering found two phases - one at lower and one at higher temperatures. Upon detailed manual inspection, we found that the angular distribution functions in these phases have the same number of peaks, but the ratio between the peak heights is different. Therefore, we could not distinguish between two phases in the peak counting method, but MDS found the differences and divided the area into two phases. Similarly, the main solid phase was also divided into several phases by MDS because the ratios between the peaks in the angular distributions are different while the positions of the peaks are the same. Comparing the hierarchical and k-means clustering, k-means seems to be more successful,

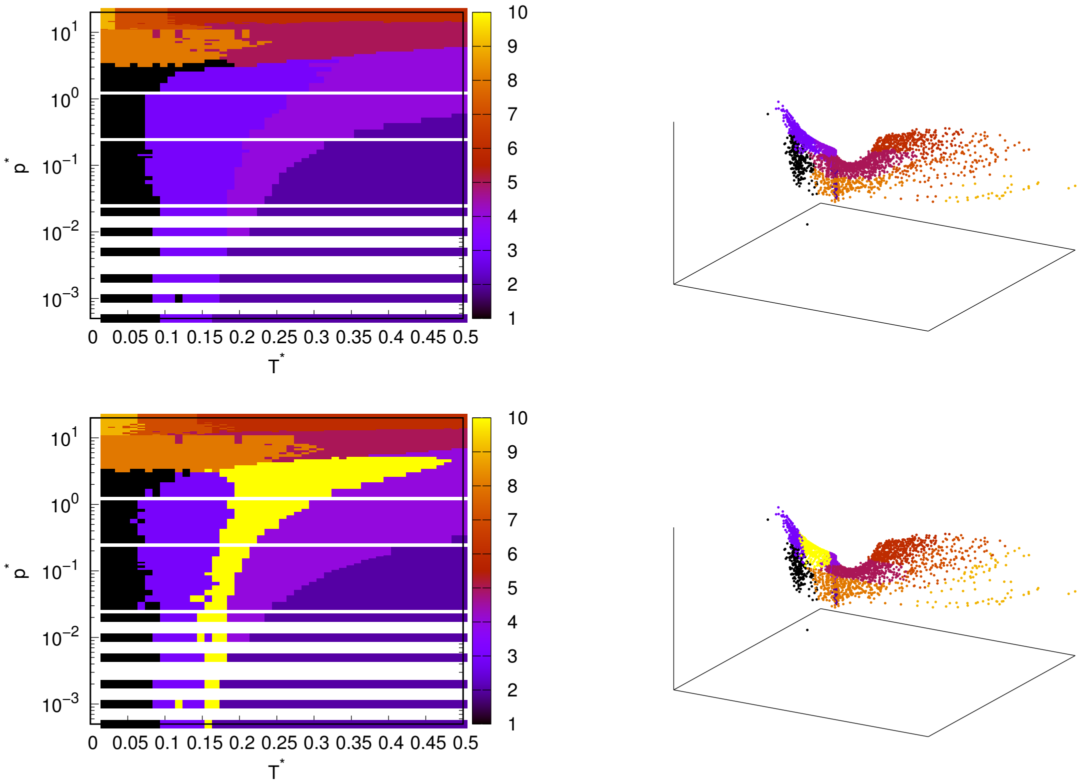

Figure S6: Pressure-temperature phase diagram of rose model with real parametrisation (left column) and projection of the same points on MDS output components (right column); obtained from angular distribution functions from MD simulations. MDS was used to decrease the dimensionality of the data, then clustering algorithms were used to cluster the data: hierarchical clustering (first line), k-means clustering (second line). DBSCAN clustering was also used but the results were not useful.

mainly because the hierarchical clustering predicted that the liquid phase is still present at too high pressure and thus in contact with the solid phase under high pressure. K-means also predicted contact between liquid and high pressure solid phase, but this contact is much smaller than when clustering with hierarchical clustering.

Isomap was also quite successful in predicting the phase diagram of the model with real parameterization. In Fig. S7 phase diagram calculated with Isomap in combination with hierarchical and k-means clustering is shown. Similar to MDS, it successfully predicted the gas and liquid phase and also the transition to the high-pressure solid phases. The solid phases are divided slightly differently when Isomap is used instead of MDS. The boundary

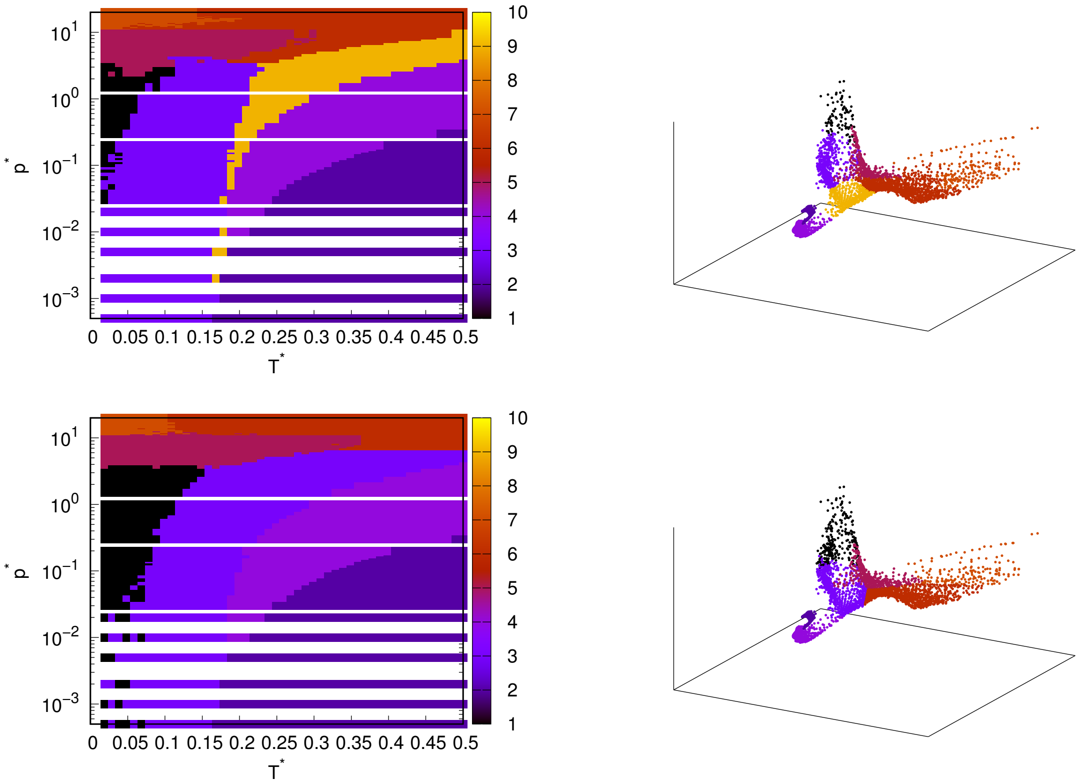

Figure S7: Pressure-temperature phase diagram of rose model with real parametrisation (left column) and projection of the same points on isomap output components (right column); obtained from angular distribution functions from MD simulations. Isomap was used to decrease the dimensionality of the data, then clustering algorithms were used to cluster the data: hierarchical clustering (first line), k-means clustering (second line). DBSCAN clustering was also used but the results were not useful.

between the horizontal solid phase at low temperatures and the hexagonal solid phase is less horizontal in the case of Isomap, and the high pressure region is also divided into fewer phases when Isomap is used instead of MDS. The division of the high pressure solid phases by isomap looks like a mixture of the division made by MDS and the division in the reference diagram. When comparing the isomap-hierarchical clustering and isomap-k-means combinations, the combination with k-means appears to be more successful, mainly because with hierarchical clustering an additional solid phase above the liquid phase was needed to separate the high-pressure solid phases from the main solid phase, whereas with k-means this region was included in the main solid phase. Another point that should be discussed

is the selection of the number of clusters when clustering with k-means and hierarchical clustering algorithms. When performing clustering, the silhouette score was calculated for clustering with different numbers of clusters, and in most cases, the number of clusters with the highest silhouette score was selected as the optimal number. However, we also used our intuition and knowledge of previous results when selecting the number of clusters. For example, if the highest silhouette score was assigned for clustering into too high or too low a number of clusters, we chose the number of clusters that had the second highest (or third highest, if necessary) silhouette score while being reasonable and consistent with intuition and previous results for our model. When calculating the phase diagram for the model with MB parameterization, the intuitively optimal number of clusters was often the one with the highest silhouette score. In contrast, in the real parameterization of the model, the highest silhouette score was often assigned to the other number of clusters that we would intuitively choose.

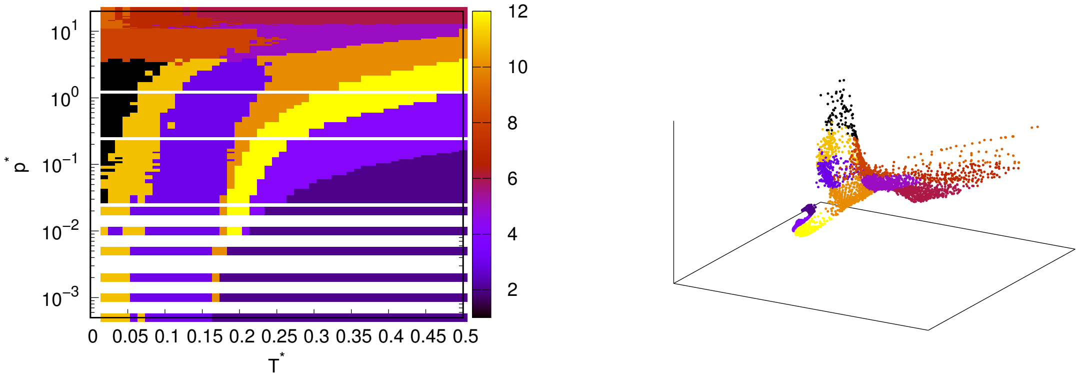

Figure S8: Pressure-temperature phase diagram of rose model with real parametrisation (left column) and projection of the same points on isomap output components (right column); obtained from angular distribution functions from MD simulations. Isomap was used to decrease the dimensionality of the data, then k-means clustering was used to cluster the data. The number of clusters is set to higher number than in Fig. S7.

In Fig. S8 phase diagram obtained with the isomap-k-means combination from the angular distribution functions is shown. The number of clusters selected here is 12, which was also the number of clusters with the highest silhouette score. The second highest silhouette score was assigned to the 7 clusters, which are shown in Fig. S7. The basic separation of

the phases is similar when using both 7 and 12 clusters, but when using 12 clusters the separation is of course more detailed. When selecting 12 clusters in k-means, the separation of the high pressure phases is very similar to MDS-k-means. There is a high pressure solid phase area at pressures from 5.0 to 10, which is divided into two parts/phases depending on the temperature. At a pressure of more than 10.0, there are also three solid phases that interchange with increasing temperature. In addition, the main solid phase is also divided into 4 phases, similar to the use of MDS. Overall, the division of the solid phases is shown in Fig. S8 is similar to that in Fig. S6. However, there is a big difference between Fig. S8 and the other phase diagrams of the real parameterization presented so far, namely the division of the liquid phase into a liquid phase with high and low density liquid phase. In conclusion, the choice of the number of clusters into which the data is divided is very important. If we had no prior knowledge of the system and selected the number of clusters based only on silhouette scores (or other separation evaluation methods), the results would not always be the best. Therefore, even when using methods that would ideally give the desired result automatically, it is very useful to have at least some prior knowledge or intuition about the system to judge whether the results are really meaningful.

Next, spectral embedding was used for dimensionality reduction (Fig. S9. Similar to the MB parameterization, this dimensionality reduction method also provided the worst results in the real parameterization case. However, in combination with the clustering algorithms, the method still predicted some phase transitions correctly. All clustering algorithms in combination with spectral embedding successfully predicted the gas and liquid phase. The methods also predicted that there are different solid phases at high pressure. However, the boundaries between the solid phases at high pressure and the liquid phase or the main hexagonal solid phase are quite different from those predicted in the reference diagram and in the diagrams obtained using other dimensionality reduction techniques.

When calculating the phase diagram of rose water model with real parameterization, t-SNE was quite successful in combination with all three clustering algorithms (Fig. S10). The parameters of t-SNE were set to the same values as when determining the phase diagram of the MB parameterization. All the main phase transitions are correctly predicted by all three combinations of the methods: These are the boundaries of the gas phase, the liquid phase and the transition to the high-pressure solid phases. The high-pressure solid phases calculated using t-SNE are similar to those calculated using isomap and MDS, and could even

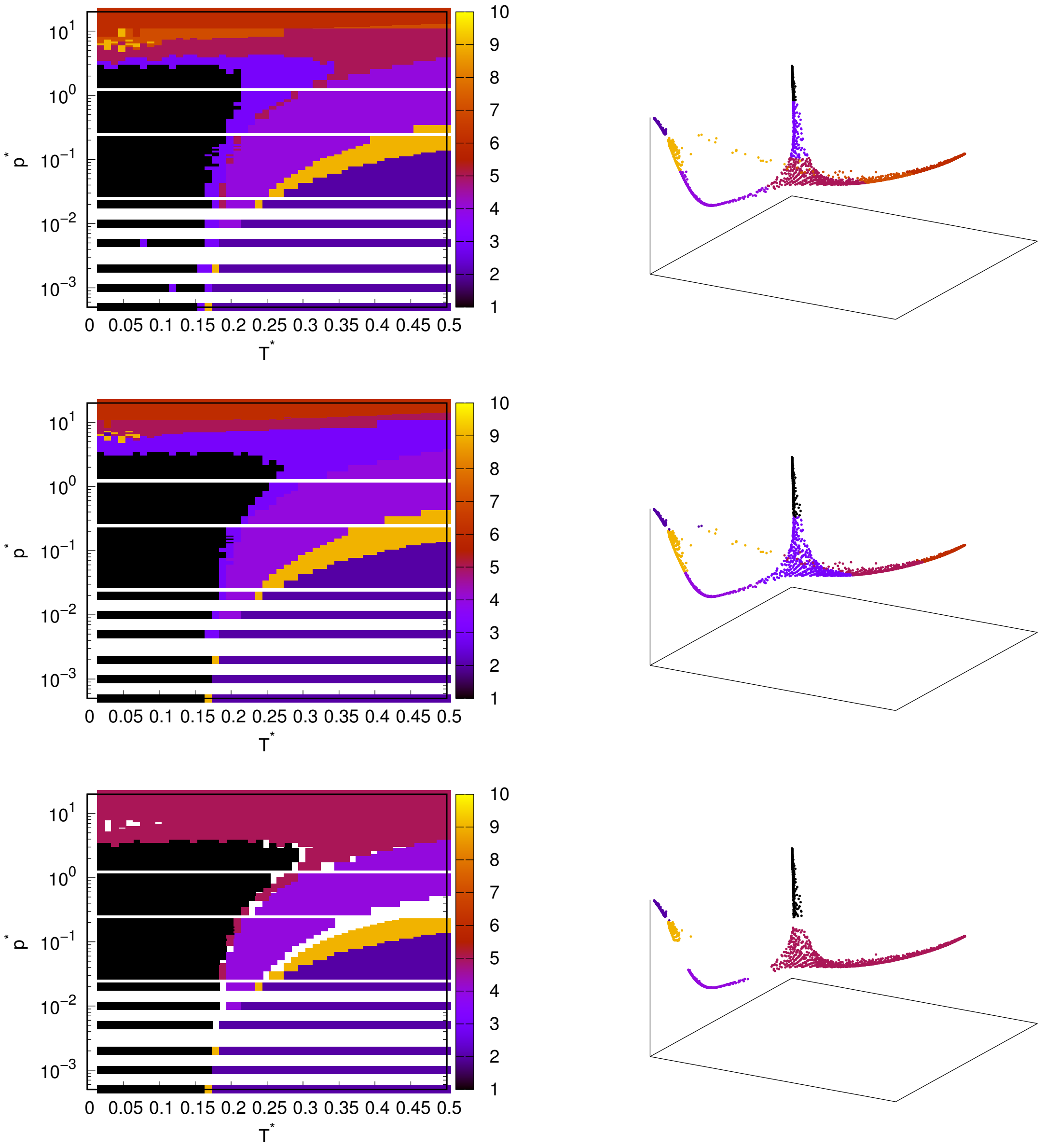

Figure S9: Pressure-temperature phase diagram of rose model with real parametrisation (left column) and projection of the same points on spectral embedding output components (right column); obtained from angular distribution functions from MD simulations. Spectral embedding was used to decrease the dimensionality of the data, then clustering algorithms were used to cluster the data: hierarchical clustering (first line), k-means clustering (second line) and DBSCAN (third line).

be more similar to the high-pressure solid phases in the reference diagram. This is because here we have one phase at a pressure of 5.0 to 10.0 and two phases at higher pressures,

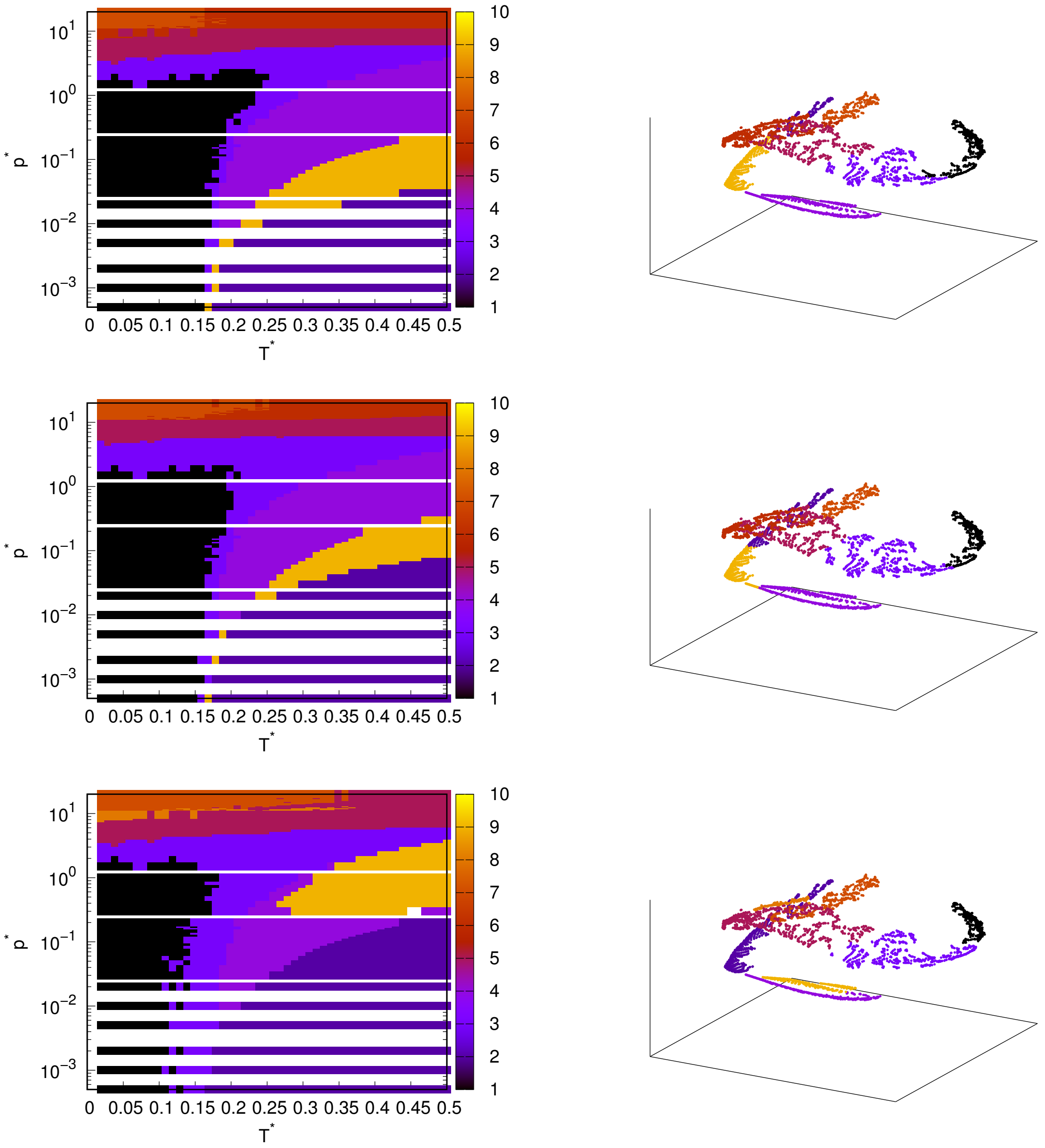

Figure S10: Pressure-temperature phase diagram of rose model with real parametrisation (left column) and projection of the same points on t-SNE output components (right column); obtained from angular distribution functions from MD simulations. T-SNE was used to decrease the dimensionality of the data, then clustering algorithms were used to cluster the data: hierarchical clustering (first line), k-means clustering (second line) and DBSCAN (third line).

one of which gradually changes into the other as the temperature rises. The agreement in the division of the high-pressure phases is particularly good between the reference diagram

and the t-SNE-k-means combination. All three clustering algorithms also divide the main hexagonal solid phase into two parts in a similar way, with one part/phase being the interior and the other being near phase transitions with other phases. The distinction into two phases is quite reasonable since the solid phase (or the part of the phase) near other phase is likely to have a more disordered structure than the inner phase (or the part of the phase), which is closer to the ideal hexagonal HB structure. Regardless of the clustering algorithm, all three diagrams appear to be very similar to each other (more so than when using other dimensionality reduction techniques). This is the result of good separation of the data points in the t-SNE output components, making the data points relatively easy to cluster by all three clustering algorithms. A small problem with phase diagrams determined with t-SNE is that the algorithms have found another liquid or gaseous phase between the liquid and gaseous phase when using k-means and hierarchical clustering. However, this phase lies in the supercritical region of the phase diagram between the liquid and gaseous phase. Therefore, it is not so wrong that the methods have shown it as a separate phase, as it represents the region where the supercritical fluid has mixed properties of liquid and gaseous phase.

Overall, when determining the phase diagram of rose water model with real parameterization, the two most successful combinations of methods are isomap-k-means and t-SNE-k-means. In a sense, t-SNE-k-means was even better than isomap-k-means, because t-SNE separates the data points more clearly and thus enables easier clustering of the data.

4. MB parametrisation - automatic approach

Fig. S13 shows the phase diagram of the rose water model with MB parameterization obtained from various thermodynamic, structural and dynamic data from MD simulations. The diagram was generated by a combination of spectral embedding and clustering algorithms. The spectral embedding results are the worst of all results obtained with different dimensionality reduction methods.

5. MB parametrisation - intelligent approach

Fig. S14 and S15 show phase diagrams determined by first separating the gas, liquid and solid phase based on the diffusion coefficient and afterwards using ML methods to separate

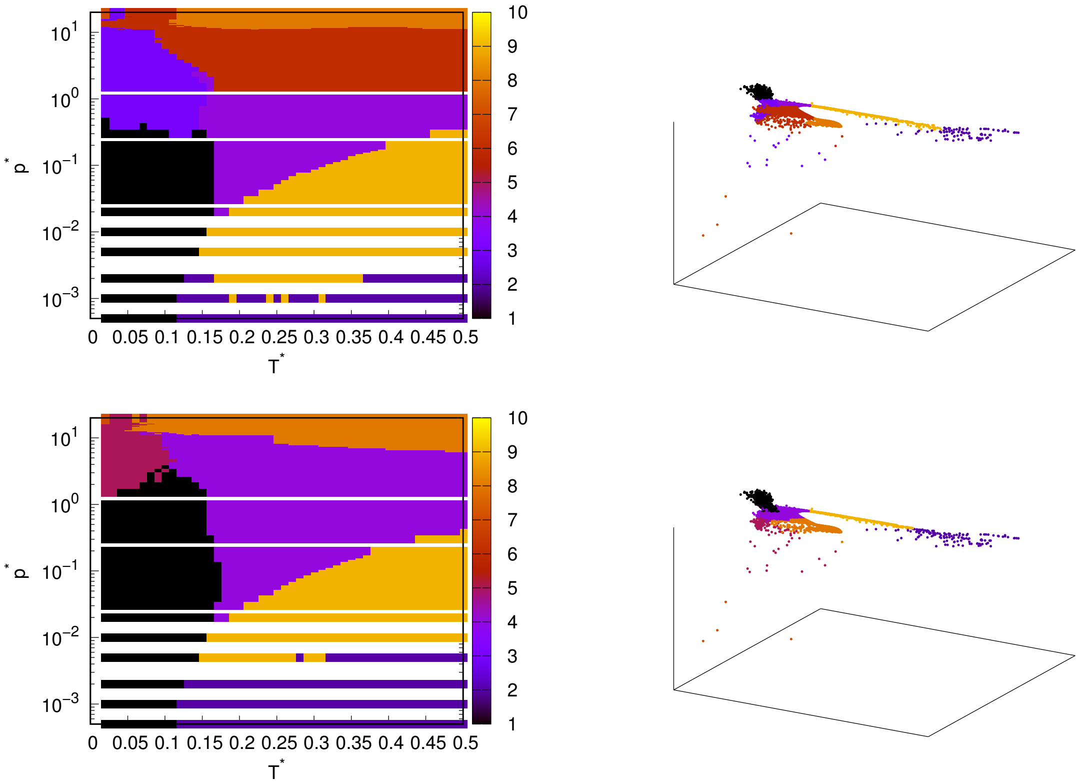

Figure S11: Pressure-temperature phase diagram of rose model with MB parametrisation (left column) and projection of the same points on MDS output components (right column); obtained from thermodynamic, structural and dynamic data from MD simulations. MDS was used to decrease the dimensionality of the data, then clustering algorithms were used to cluster the data: hierarchical clustering (first line), k-means clustering (second line). DBSCAN clustering was also used but the results were not useful.

different solid phases.

6. Real parametrisation - automatic approach

Table S2 shows fractions of agreement between phase diagrams of rose model with real parametrisation determined by ML methods and reference phase diagram. The input data for ML methods is combination of thermodynamic, dynamic and structural quantities obtained from simulations.

The phase diagram of rose model with real parameterization was determined from simu-

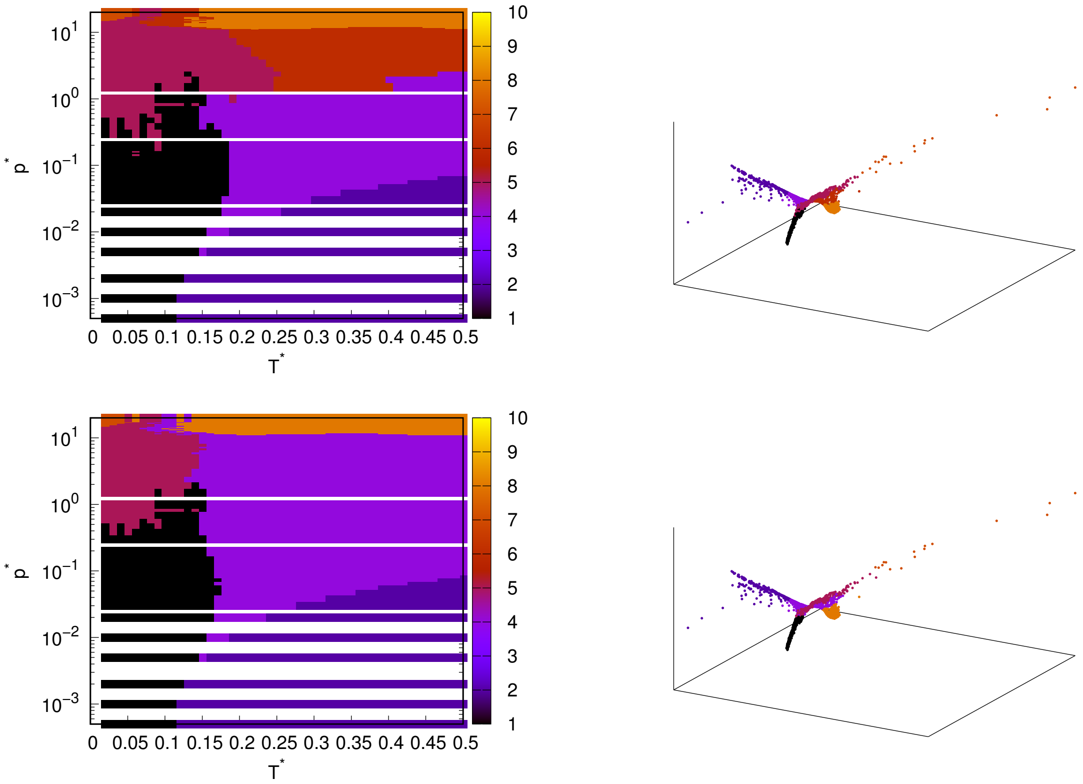

Figure S12: Pressure-temperature phase diagram of rose model with MB parametrisation (left column) and projection of the same points on isomap output components (right column); obtained from thermodynamic, structural and dynamic data from MD simulations. Isomap was used to decrease the dimensionality of the data, then clustering algorithms were used to cluster the data: hierarchical clustering (first line), k-means clustering (second line). DBSCAN clustering was also used but the results were not useful.

lation data in the same way as for the MB parameterization. First, we tried to determine it directly from all simulation quantities in the entire phase space (Fig. S16, S17, S18, S19). Similar to the MB parameterization, the results were mixed and the agreement with the reference diagram and the phase diagrams from the angular distribution functions was inconsistent. Some of the most important phase transitions were successfully predicted, while others were not. Furthermore, in many cases additional phase transitions that probably do not exist were predicted by the algorithms. All phase diagrams of rose model with the real parameterization obtained with the same methods used for the MB parameterization are shown in the Supplementary Information. A detailed discussion of these diagrams is

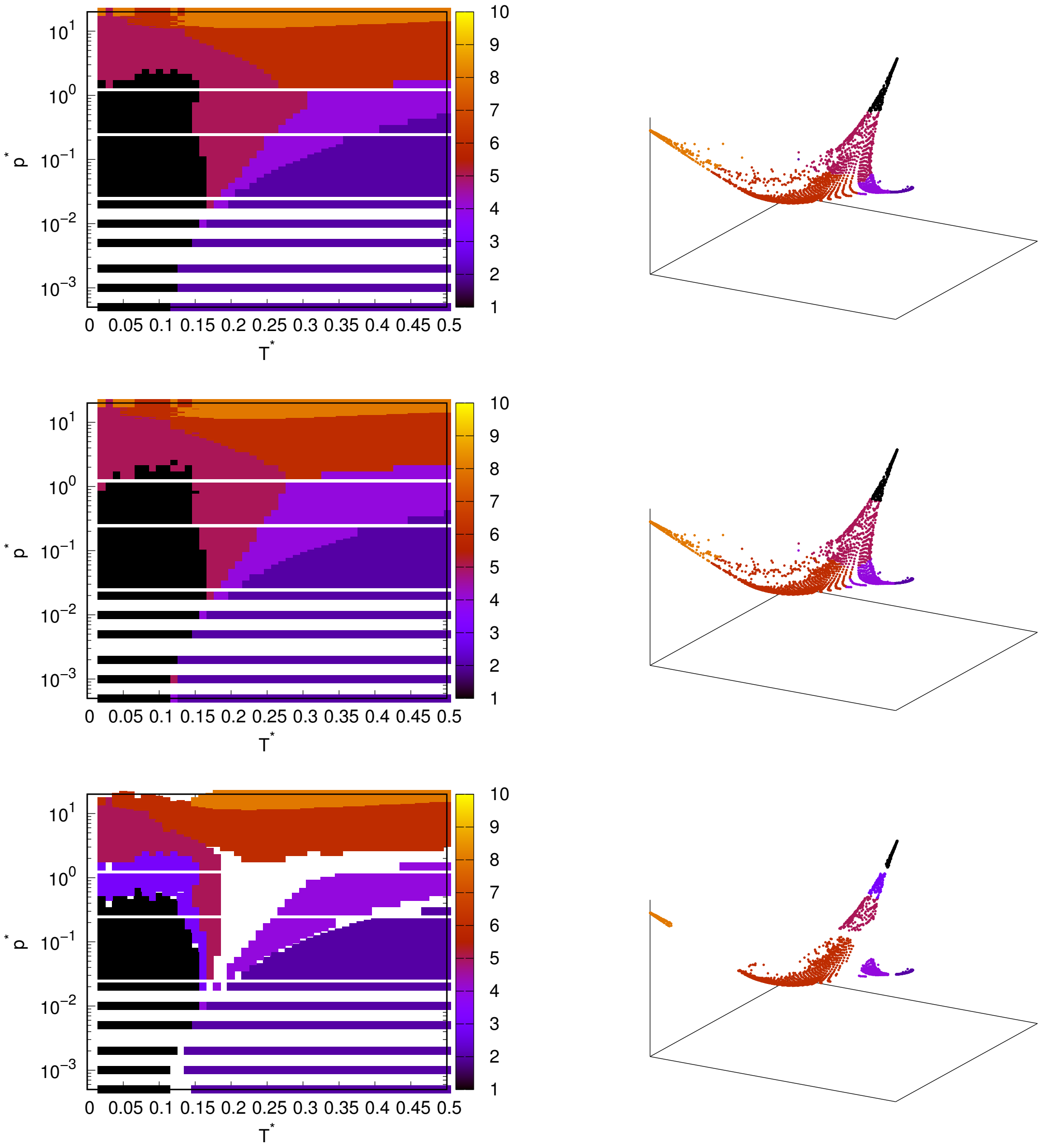

Figure S13: Pressure-temperature phase diagram of rose model with MB parametrisation (left column) and projection of the same points on spectral embedding output components (right column); obtained from thermodynamic, structural and dynamic data from MD simulations. Spectral embedding was used to decrease the dimensionality of the data, then clustering algorithms were used to cluster the data: hierarchical clustering (first line), k-means clustering (second line) and DBSCAN (third line).

omitted as they do not add any value other than the realisation that the direct/automatic use of different simulation data in dimensionality reduction methods followed by cluster-

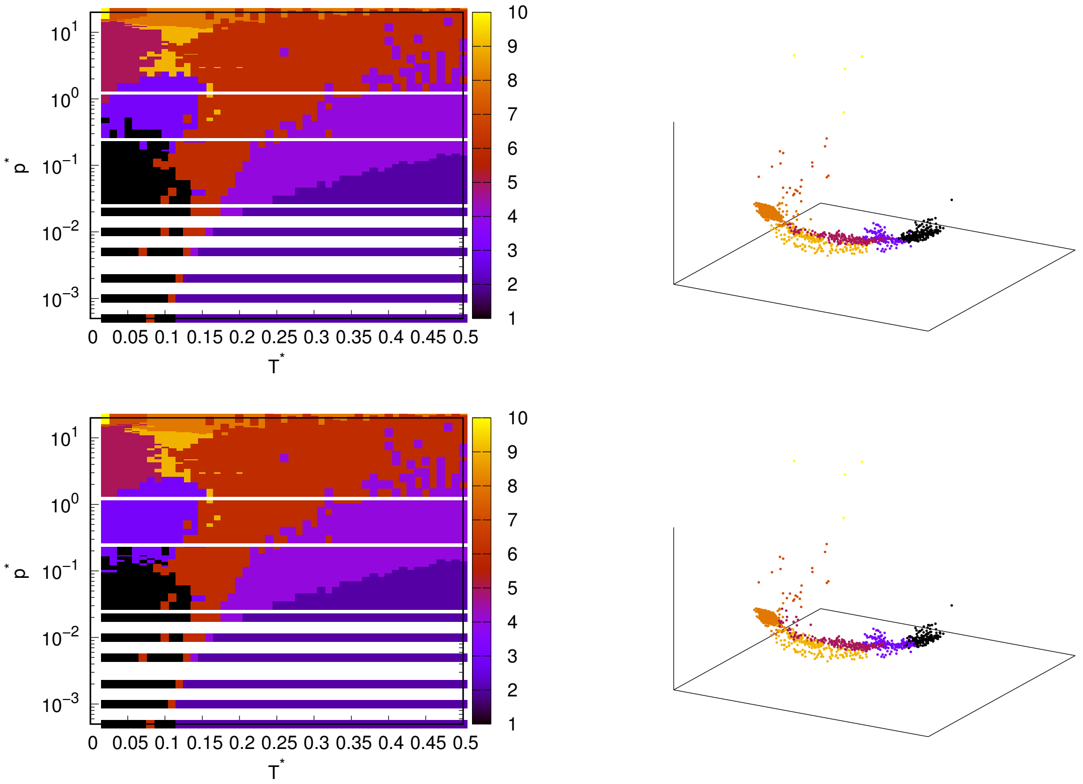

Figure S14: Pressure-temperature phase diagram of rose model with MB parametrisation (left column) and projection of the points of solid phases on MDS output components (right column); obtained from thermodynamic, structural and dynamic data from MD simulations. First DBSCAN and diffusion coefficient were used to determine solid, liquid and gas phase, then MDS in combination with clustering algorithms (hierarchical clustering (first line), k-means clustering (second line). DBSCAN clustering was also used but the results were not useful.

ing algorithms does not give the best results, but still correctly predicts some of the phase transitions. One of the reasons that an automatic approach, where we simply take all the data into the algorithm, is not optimal is that the differences between the different phases vary in size. For example, the difference between the solid and the gaseous phase is large, while the difference between two neighbouring solid phases is small. Therefore, if both phase transitions are determined in the same step, we may miss the transition between the solid phases phase because the two solid phases are relatively similar to each other compared to the gaseous phase. To separate the solid phases more efficiently, it is therefore advisable to

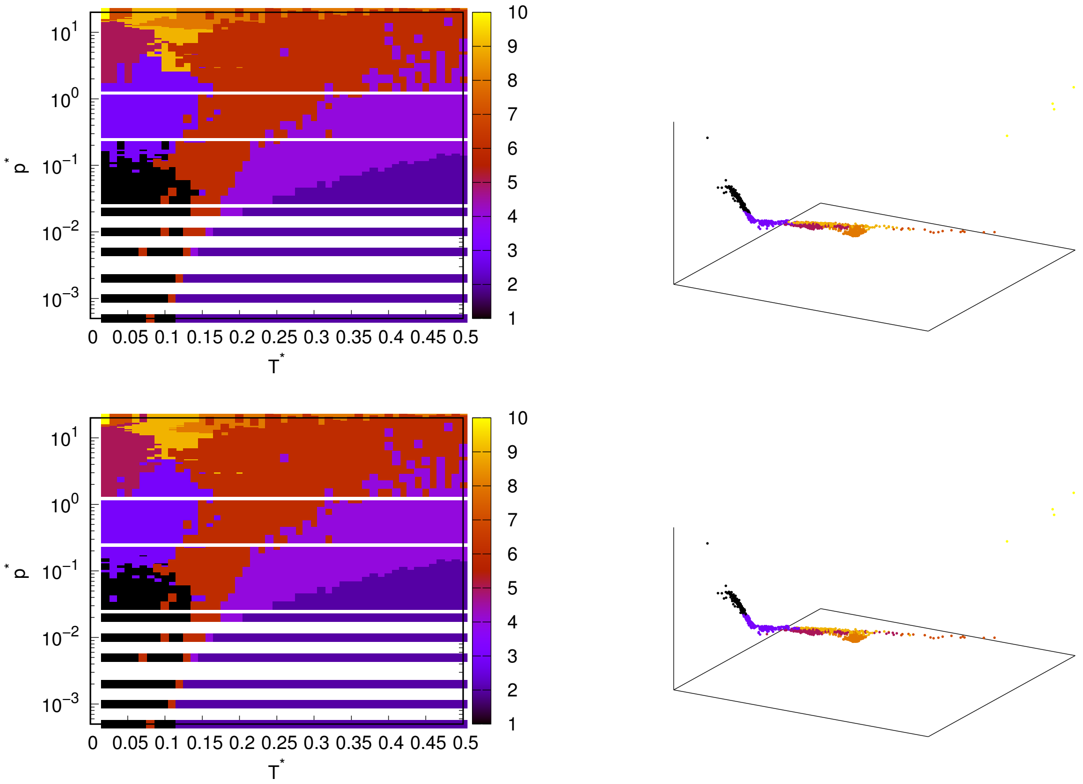

Figure S15: Pressure-temperature phase diagram of rose model with MB parametrisation (left column) and projection of the points of solid phases on isomap output components (right column); obtained from thermodynamic, structural and dynamic data from MD simulations. First DBSCAN and diffusion coefficient were used to determine solid, liquid and gas phase, then isomap in combination with clustering algorithms (hierarchical clustering (first line), k-means clustering (second line). DBSCAN clustering was also used but the results were not useful.

separate the more "drastic" phase transitions and the "mild" phase transitions.

7. Real parametrisation - intelligent approach

For rose model with MB parameterization, the approach of first separating the solid, liquid and gaseous phases based on the diffusion coefficient and then further subdividing the solid phases based on other simulation data showed a significant improvement over processing all quantities from the simulations in one step. Here we have used the same

Dimensionality reduction	Clustering algorithm	Fraction of agreement
Isomap	K-means	0.633
Isomap	Hierarchical	0.557
MDS	K-means	0.553
MDS	Hierarchical	0.603
t-SNE	K-means	0.790
t-SNE	Hierarchical	0.788
t-SNE	DBSCAN	0.640
Spectral Em.	K-means	0.704
Spectral Em.	Hierarchical	0.675
Spectral Em.	DBSCAN	0.678

Table S2: Fraction of agreement between the phase diagram of rose model with real parametrisation calculated with each combination of methods and the reference diagram. The diagrams are derived from combination of thermodynamic, dynamic and structural data.

process again for the model with real parameterization.

Table S3 shows fractions of agreement between phase diagrams of rose model with real parametrisation determined by ML methods and reference phase diagram. Here the gas, liquid and solid phases were first separated based on diffusion using DBSCAN, then solid phases were separated using combination of ML methods. The input data for ML methods is combination of thermodynamic, dynamic and structural quantities obtained from simulations.

In Fig. S20, S21 and S22 the phase diagrams of rose water model with real parametrisation are shown. The diagrams are obtained by a two-step method - the first step is the separation of solid, liquid and gaseous phase based on the diffusion coefficient, while the second step is the separation of the solid phase based on different simulation data (as in the MB parameterization). The separation into solid, liquid and gaseous phases is shown in all three figures (Fig. S20, S21 and S22) is the same and agrees very well with the reference diagram. When the phase separation was performed for the MB parameterization of the model, three consecutive DBSCANs were required (in the first DBSCAN the gas phase was

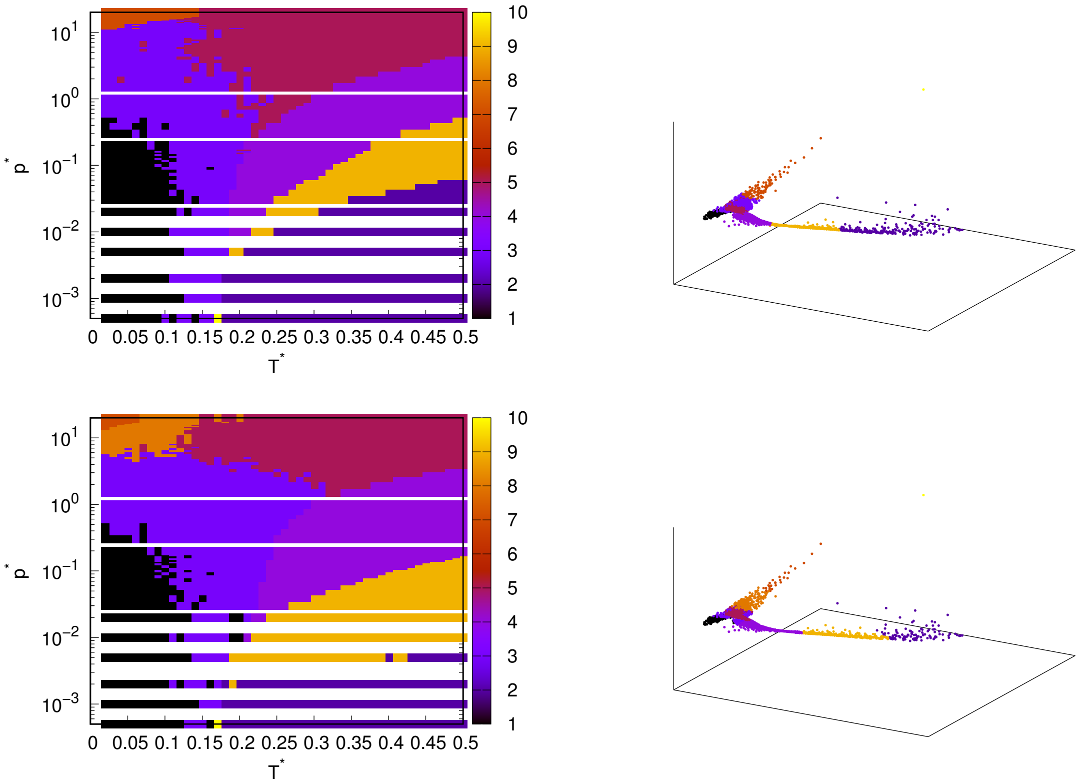

Figure S16: Pressure-temperature phase diagram of rose model with real parametrisation (left column) and projection of the same points on MSD output components (right column); obtained from thermodynamic, structural and dynamic data from MD simulations. MSD was used to decrease the dimensionality of the data, then clustering algorithms were used to cluster the data: hierarchical clustering (first line), k-means clustering (second line). DBSCAN clustering was also used but the results were not useful.

separated, in the second the supercritical-ish liquid phase and in the third DBSCAN the liquid phase was separated from the solid phases). On the other hand, only two consecutive DBSCANs were required for the phase separation of the model with real parameterization (the first separated the gas phase and the second separated the liquid phase). Overall, this method of phase separation with DBSCAN based on the diffusion coefficient is quite accurate and robust. It could also be used with the data from MC simulations, using a pseudo-diffusion coefficient instead of the diffusion coefficient. The method would work because the absolute values of the diffusion coefficient are not important and only the relative differences between the diffusion coefficients are important. In addition, the diffusion co-

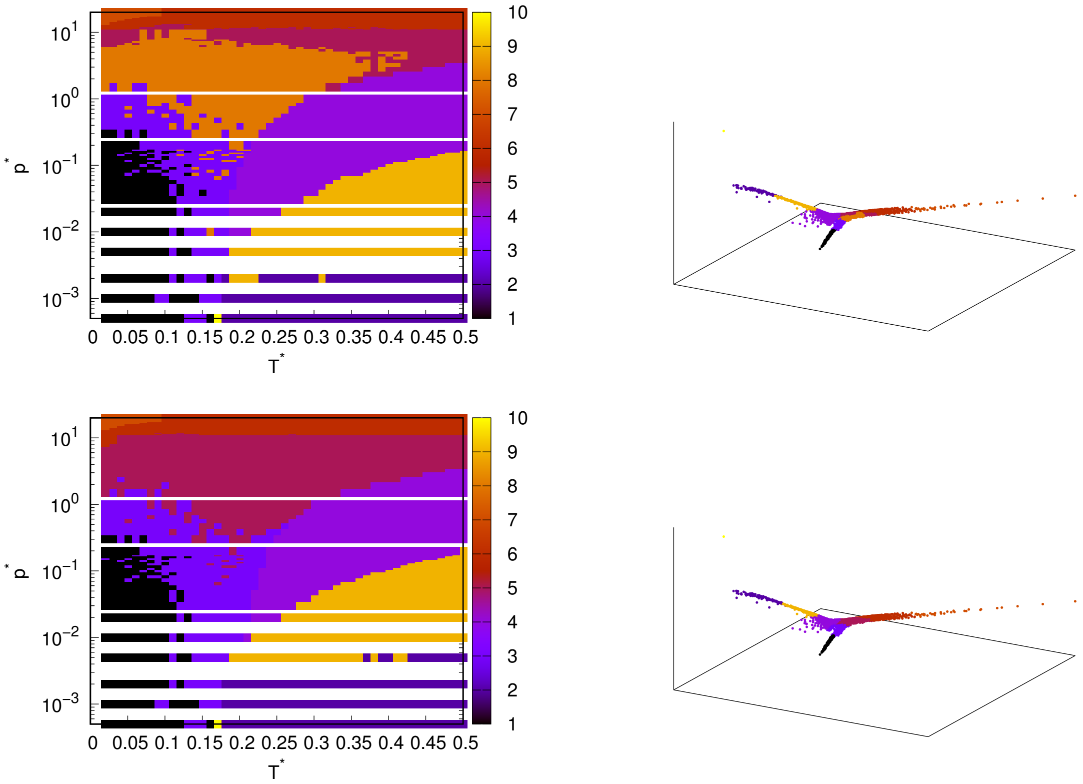

Figure S17: Pressure-temperature phase diagram of rose model with real parametrisation (left column) and projection of the same points on isomap output components (right column); obtained from thermodynamic, structural and dynamic data from MD simulations. Isomap was used to decrease the dimensionality of the data, then clustering algorithms were used to cluster the data: hierarchical clustering (first line), k-means clustering (second line). DBSCAN clustering was also used but the results were not useful.

efficient values are normalized before the DBSCAN anyway. Moving to separation of the solid phases. In the MB parameterization, t-SNE was very successful in dimensionality reduction because the points in the new 3D space were well separated and thus clustering of these points was easy. Unfortunately, this is not the case with the real parameterization of the model. In Fig. S20 shows the solid state points in the t-SNE component space. It is obvious that the separation into clusters is not as good as in the MB parameterization. Consequently, clustering the points with different clustering algorithms is also not easy and the results are not as solid as with the MB parameterization. The DBSCAN locates some clusters with higher density, but when manually checking the position of these clusters in

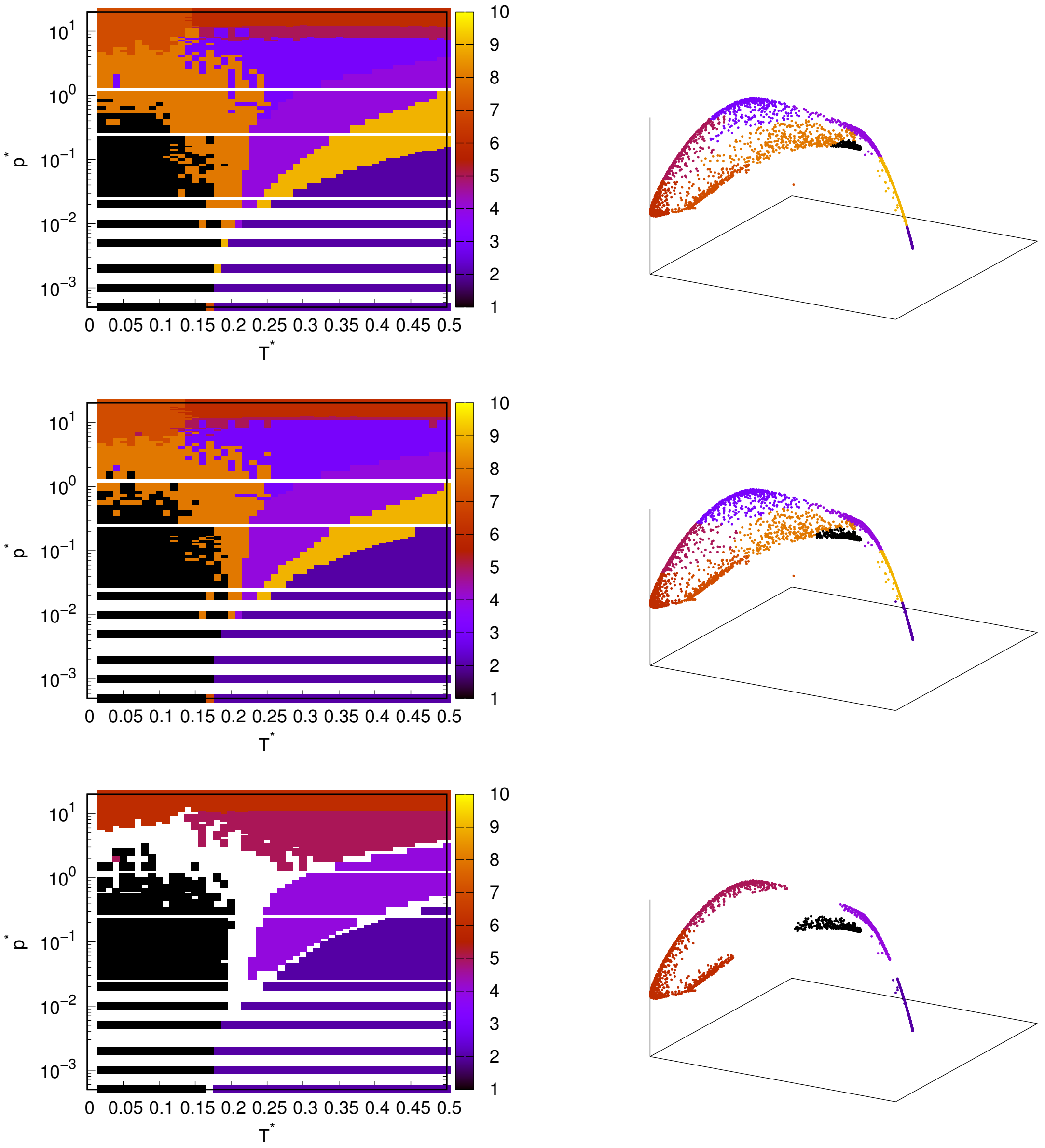

Figure S18: Pressure-temperature phase diagram of rose model with real parametrisation (left column) and projection of the same points on spectral embedding output components (right column); obtained from thermodynamic, structural and dynamic data from MD simulations. Spectral embedding was used to decrease the dimensionality of the data, then clustering algorithms were used to cluster the data: hierarchical clustering (first line), k-means clustering (second line) and DBSCAN (third line).

3D space, they are usually not clearly separated from the rest of the point and it seems that the clusters are just randomly selected parts of a larger cluster. Therefore, clustering data

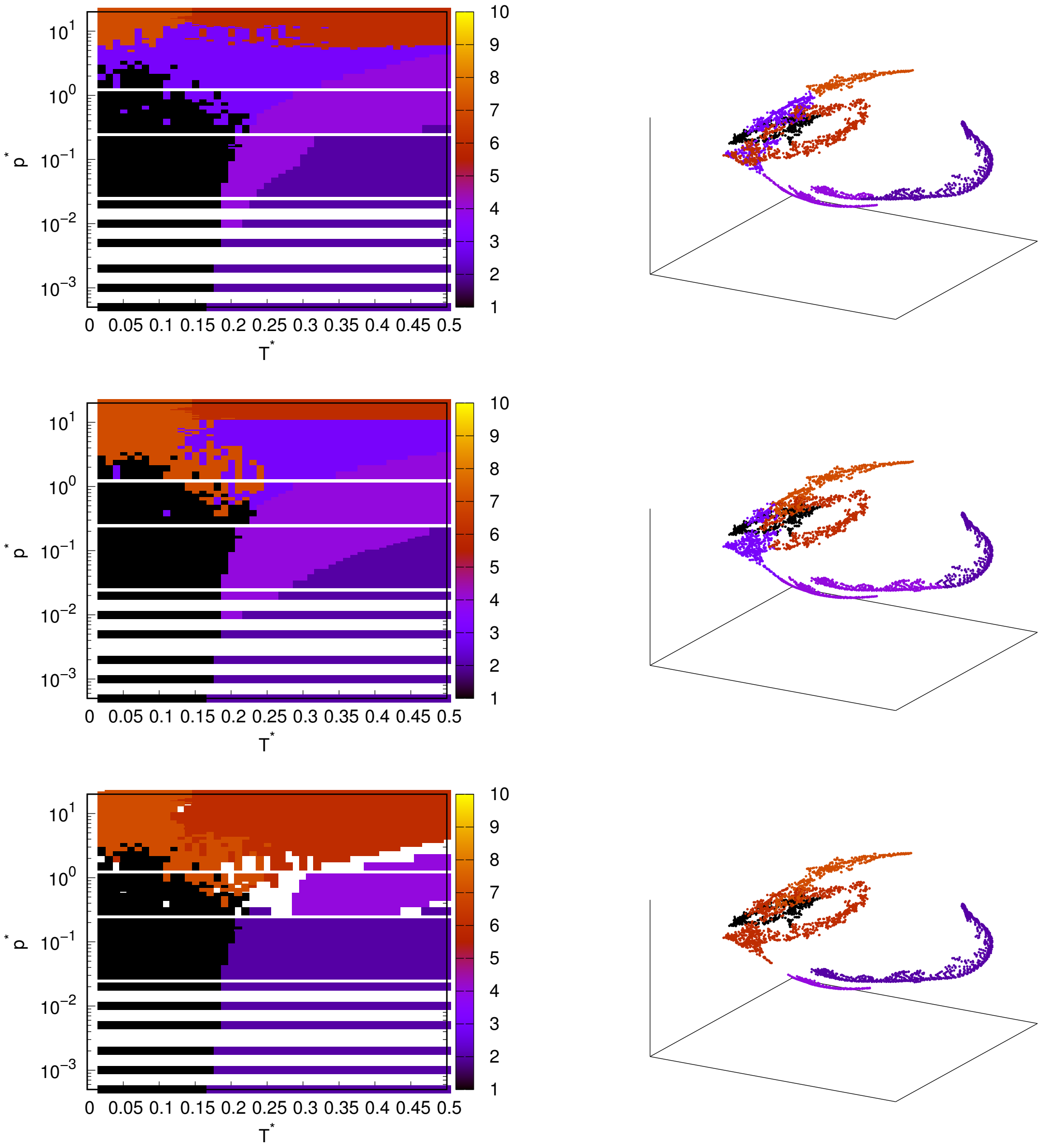

Figure S19: Pressure-temperature phase diagram of rose model with real parametrisation (left column) and projection of the same points on t-SNE output components (right column); obtained from thermodynamic, structural and dynamic data from MD simulations. T-SNE was used to decrease the dimensionality of the data, then clustering algorithms were used to cluster the data: hierarchical clustering (first line), k-means clustering (second line) and DBSCAN (third line).

with the t-SNE-DBSCAN combination is rather unsuccessful. The k-means and hierarchical clustering result in similar phase diagrams, as the phases are the same, but the boundaries

Dimensionality reduction	Clustering algorithm	Fraction of agreement
Isomap	K-means	0.664
Isomap	Hierarchical	0.621
MDS	K-means	0.570
MDS	Hierarchical	0.584
t-SNE	K-means	0.665
t-SNE	Hierarchical	0.674
t-SNE	DBSCAN	0.606

Table S3: Fraction of agreement between the phase diagram of rose model with real parametrisation calculated with each combination of methods and the reference diagram. The diagrams are derived from combination of thermodynamic, dynamic and structural data using the procedure described above. The gas, liquid and solid phases are separated based on diffusion by DBSCAN, while different solid phases are separated using different combinations of methods.

between them are slightly different. The number of clusters was chosen based on the highest silhouette score. When comparing these phase diagrams with the diagrams obtained from the angular distributions, there are some differences and some similarities. The diagrams are similar in that the main hexagonal solid phase is divided into two parts - the low and high temperature one. This division is valid as it correlates with three-fold symmetry factor, for example, as the hexagonal HB network has more empty hexagonal vacancies in the low temperature part than in the high temperature part. Another similarity of these diagrams to diagrams from angular distributions is that the high-pressure solid phases are separated from the main solid phase(s), but the boundaries between the different high-pressure solid phases are different.

Next, we used MDS for dimensionality reduction (Fig. S21). Here, k-means and hierarchical clustering were used for clustering the data points. We also tried DBSCAN, but the results were not useful. The phase diagrams obtained with MDS are quite different from those obtained with t-SNE, and even the diagrams which are both obtained with MDS are quite different from each other. Both MDS-k-means and MDS-hierarchical clustering predict a separation of the main hexagonal phase into a low-temperature and a high-temperature

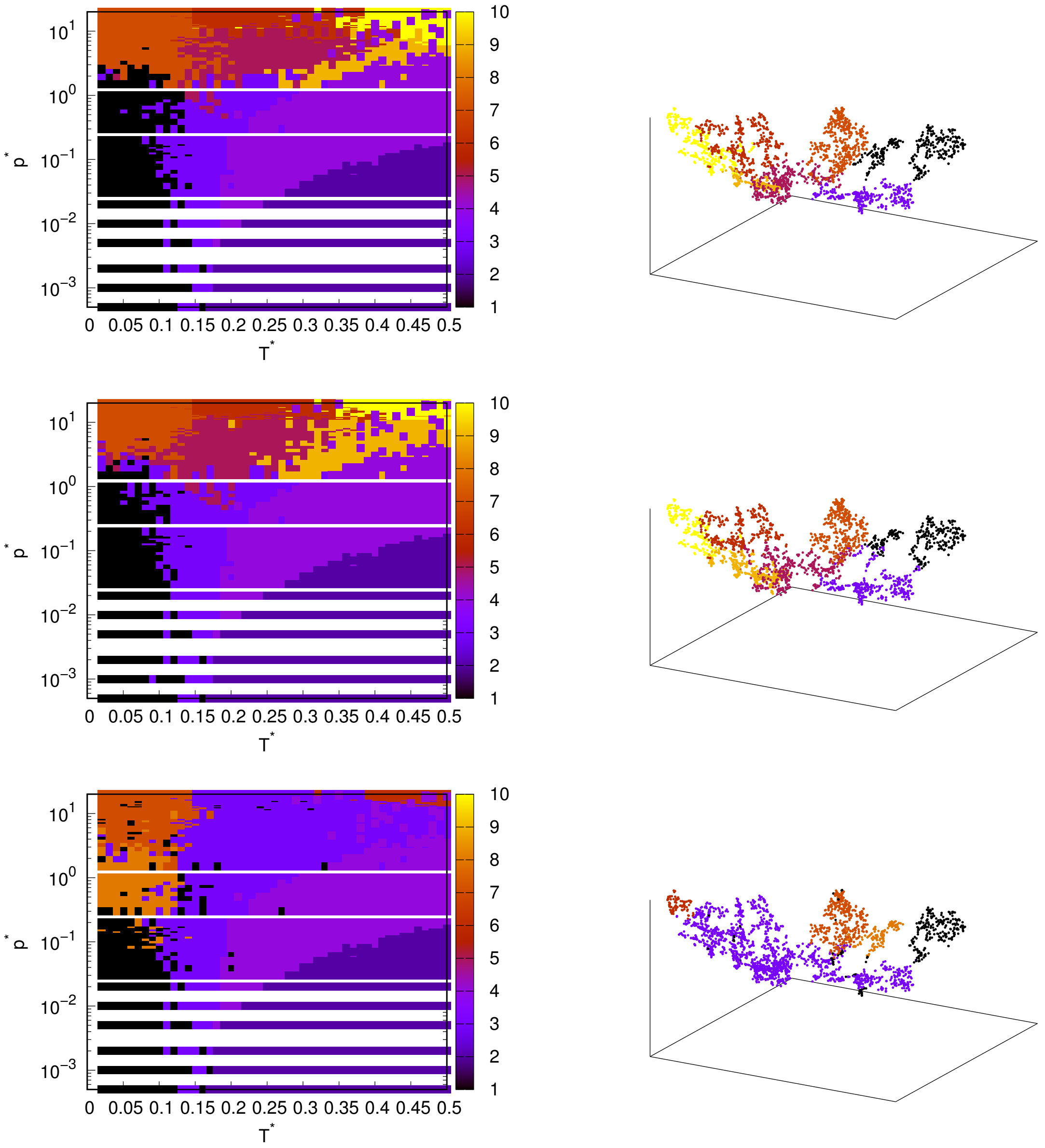

Figure S20: Pressure-temperature phase diagram of rose model with real parametrisation (left column) and projection of the points of solid phases on t-SNE output components (right column); obtained from thermodynamic, structural and dynamic data from MD simulations. First DBSCAN and diffusion coefficient were used to determine solid, liquid and gas phase, then T-SNE in combination with clustering algorithms (hierarchical clustering (first line), k-means clustering (second line) and DBSCAN (third line)) were used to separate solid phases.

part, but this separation is more diffuse and not as clear as with t-SNE. At high pressure,

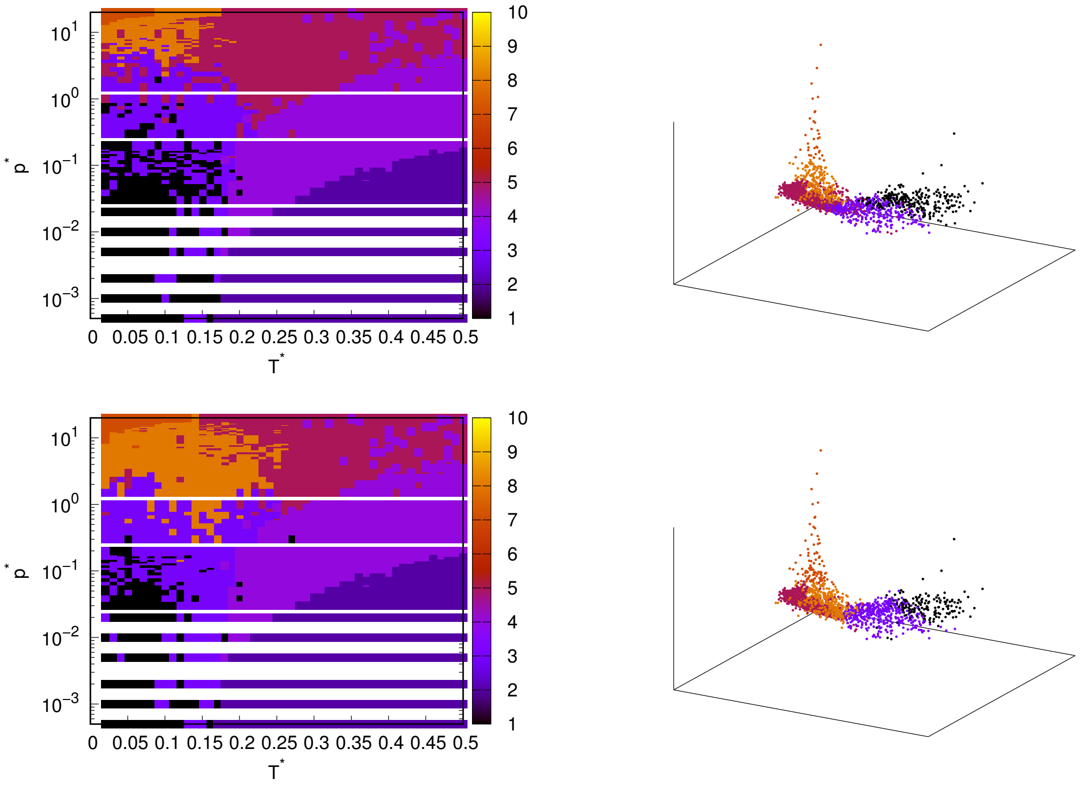

Figure S21: Pressure-temperature phase diagram of rose model with real parametrisation (left column) and projection of the points of solid phases on MDS output components (right column); obtained from thermodynamic, structural and dynamic data from MD simulations. First DBSCAN and diffusion coefficient were used to determine solid, liquid and gas phase, then MDS in combination with clustering algorithms (hierarchical clustering (first line), k-means clustering (second line). DBSCAN clustering was also used but the results were not useful.

both clustering algorithms predict a phase at pressure 20.0 and a temperature close to zero. On the other hand, at higher temperatures, they only predict a solid phase in the range from the liquid phase to pressure 20.0.

The phase diagrams obtained from different simulation data using isomap and clustering algorithms look like a mixture of the diagrams obtained with t-SNE and MDS. The diagrams are shown in Fig. S22. Both clustering algorithms cluster the data in a similar way. While the predicted phases are the same, the boundaries between them differ slightly but are still very similar. The number of clusters is selected based on the highest silhouette score.

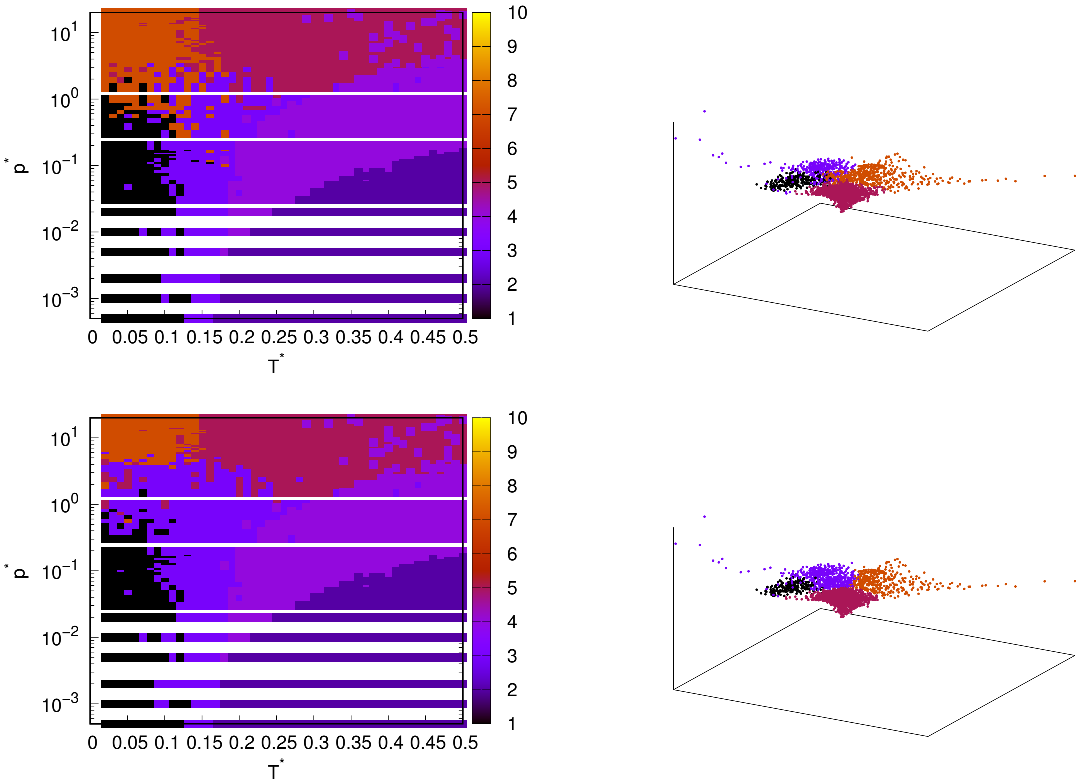

Figure S22: Pressure-temperature phase diagram of rose model with real parametrisation (left column) and projection of the points of solid phases on isomap output components (right column); obtained from thermodynamic, structural and dynamic data from MD simulations. First DBSCAN and diffusion coefficient were used to determine solid, liquid and gas phase, then isomap in combination with clustering algorithms (hierarchical clustering (first line), k-means clustering (second line). DBSCAN clustering was also used but the results were not useful.

Both clustering algorithms in combination with the isomap predict the division of the main hexagonal solid phase into low and high temperature part, this separation is similar to that using t-SNE. In addition, the phase space above a pressure of about 2.0 is also separated into two phases based on temperature. The separated solid phase in the upper left corner of the diagrams is similar to the diagrams obtained with t-SNE.

Overall, it is difficult to say which combination of dimensionality reduction and clustering algorithm gave the best result in separating solid phases of the model with real parameterization based on different structural, thermodynamic and dynamic quantities. The results of

all dimensionality reduction methods differ significantly from each other and also from the results obtained using angular distribution functions. Moreover, the boundaries between the different predicted solid phases are very diffuse in the case of the real parameterization of the model, while the boundaries were better defined and more distinct in the case of the MB parameterization. Another surprise was that the separation of the data points with t-SNE was much less good in the case of the real parameterization compared to the MB parameterization. The reason for the more difficult distinction between solid phases is of course the parameterization of the model, which in the case of the real parameterization results in the properties of the model/system being less divers across the different conditions. A major difference between the parameterization of the model is that the MB parameterization has two different characteristic radial distances, while the real parameterization has only one. This difference obviously reduces the variety of phases considerably.

There is also the possibility that the rose model with real parameterization has only a single solid phase and that the phases we have found are just different parts of the same phase with slightly different properties. There are many indications that this could be the case. First, if we look at the snapshots of the system under different conditions (Fig. S4 and S5), it looks like the (manually determined) solid phases are basically the variations of the hexagonal dense packing. Starting with phases (5), (6) and (7), they are indeed hexagonal dense packings with slight differences that are barely visible to the naked eye. Furthermore, phase (1) is indeed also a hexagonal dense packing, but with a lot of vacancies. Under different conditions, phase (1) has a different number of voids. However, if all voids were filled, it would be a hexagonal dense packing. This is a consequence of two things - the characteristic radial distance and the formation of "half" bonds. Because of the one characteristic distance, this distance will always be favoured (unless high pressure prevents it), and because of the half bonds, the molecule will always try to adopt the most favourable orientation, which is independent of the orientation of the neighbouring molecules. Consequently, a molecule in an ideal hexagonal network of hydrogen bonds has the energy $3/2 * \epsilon_{HB} + 3/2 * \epsilon_{LJ}$, whereas in a hexagonal dense packing, which in this case actually corresponds to the ideal hexagonal HB network in which all vacancies are filled, the molecules of the HB network have the same energy and the molecules in the vacancies have the energy $3/2 * \epsilon_{HB} + 6/2 * \epsilon_{LJ}$. The ideal hexagonal dense packing is therefore more favourable in terms of energy and enthalpy (due to the smaller volume). Nevertheless, both structures are basically the same, only

with a different degree of filled hexagonal vacancies. So there can only be one solid phase, but depending on the conditions (temperature and pressure) this phase can be more or less disordered and with more or less vacancies filled. As we have already mentioned, the boundaries between the different solid phases determined by the different methods presented in this work are much less defined and distinguishable for the real parameterization than in the MB parameterization. This suggests that the points in the phase space have more similar properties and may therefore be more difficult to distinguish. If the real parameterization indeed has only one solid phase, the methods used to find different phases have merely split the one phase into parts with different properties.
